# Systematic Proteomic Characterization of EV‐A71‐Infected Mice Identifies Dynamic Molecular Changes and Therapeutic Targets

**DOI:** 10.1002/mco2.70668

**Published:** 2026-04-02

**Authors:** Wanjun Peng, Qiaochu Wang, Binbin Zhao, Lihong Zhang, Jing Wu, Xiaohui Wei, Na Rong, Zhaohua Wang, Kaihui Liu, Jiangfeng Liu, Juntao Yang, Jiangning Liu

**Affiliations:** ^1^ NHC Key Laboratory of Human Disease Comparative Medicine Beijing Key Laboratory for Animal Models of Emerging and Remerging Infectious Diseases Institute of Laboratory Animal Science Chinese Academy of Medical Sciences and Comparative Medicine Center Peking Union Medical College Beijing China; ^2^ State Key Laboratory of Common Mechanism Research for Major Diseases Department of Biochemistry and Molecular Biology Institute of Basic Medical Sciences Chinese Academy of Medical Sciences School of Basic Medicine Peking Union Medical College Beijing China

**Keywords:** antiviral, enterovirus A71 (EV‐A71), multiple‐omics, phosphoproteomics, proteomics, transcriptomic

## Abstract

Enterovirus A71 (EV‐A71) is recognized as the primary causative agent of hand, foot, and mouth disease (HFMD) and is prevalent worldwide. However, the precise pathogenic mechanisms of EV‐A71 remain unclear, and specific drugs targeting it have yet to be successfully developed. To explore the mechanisms underlying EV‐A71 pathogenesis and to identify potential therapeutic opportunities, we performed a comprehensive proteogenomic characterization of muscle tissues from BALB/c mice infected with EV‐A71, integrating transcriptomic, proteomic, and phosphoproteomic analyses. Our results showed that phagosome, complement, and coagulation cascade pathway‐related molecules were activated, and the expression of cell growth‐related molecules was downregulated. Concurrently, a rapid activation of the neutrophil extracellular trap pathway was observed at the protein level. Additionally, we mapped the global phosphorylation profiles to dysregulated kinases, predicting 32 drugs corresponding to 27 kinases. We found that kinase inhibitors have antiviral activity in vitro; vandetanib, nintedanib, dasatinib, avitinib, and nilotinib can inhibit virus replication in mice to some extent. Overall, this study provides a multi‐omics resource for elucidating EV‐A71‐induced alterations in target tissues and for linking omics‐based target discovery with drug screening and functional validation, providing new insights into both pathogenesis and therapeutic exploration.

## Graphical abstract

Time‐resolved multi‐omics of EV‐A71‐infected BALB/c mice uncovered dynamic host responses, revealing rapid neutrophil extracellular traps (NETs) activation as a key event. Screened drugs (kinase inhibitors) suppressed viral replication in vitro and in vivo, suggesting the promise of targeting host phosphorylation networks as a viable antiviral strategy.

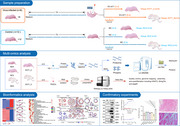

## 1 Introduction

Hand, foot, and mouth disease (HFMD) is an infectious disease caused by a variety of pathogens; enterovirus A71 (EV‐A71) is the main serotype that causes severe cases [1]. Most HFMD cases occur in the summer, and patients are mostly infants and young children who present with fever, oral ulcers, and rashes on the hands, feet, and buttocks. Although most cases are self‐limiting, severe cases can progress to neurological complications, such as aseptic meningitis, pulmonary edema, encephalitis, and acute flaccid paralysis [2, 3, 4]. In 2008, a large HMFD epidemic occurred in China, with more than 480,000 cases and 126 deaths reported. The number of affected individuals continued to increase between 2008 and 2015, with 3300 deaths and up to a 1.1% incidence of severe disease [5]. Following the launch of the monovalent EV‐A71 vaccine, the prevalence of EV‐A71 has been controlled to a certain extent, and the number of cases has decreased annually but still accounts for a large proportion of HFMD cases [6]. To date, the pathogenic mechanism of EV‐A71 has not been elucidated, and there are no effective therapeutic drugs available.

The transcriptome, proteome, and phosphoproteome are powerful tools for elucidating the mechanisms of viral pathogenesis. To date, there have been few proteomic analyses of EV‐A71 using cells as the experimental model [7]. H. Shi et al. analyzed the proteomic and metabolomic data from EV‐A71‐infected rhabdomyosarcoma (RD) cells and reported 26 differentially expressed proteins (DEPs) [8]. Systematic and integrative multi‐omics investigations of in vivo EV‑A71 infection remain lacking. Multi‐omics analysis is one of the most powerful tools for screening drug targets. By correlating the phosphorylation profiles of dysregulated signaling pathways with known drugs and compounds targeting these pathways, it is feasible to screen for small‐molecule inhibitors against key pathways such as NF‐κB, MAPK, and PI3K/AKT. These inhibitors may exert dual antiviral functions: on one hand, they can indirectly suppress viral replication and assembly by blocking the virus's exploitation of host signaling pathways; on the other hand, they may modulate the host inflammatory response through regulation of these pathways. The precise mechanisms of action and therapeutic efficacy of these compounds warrant further investigation. Similar strategies have been applied in other viral and disease contexts. M. Bouhaddou et al. identified and tested 68 drugs and found that several drugs had anti‐COVID‐19 activity [9]. A. Stukalov et al. identified potential targets for treating COVID‐19 through multi‐omics analyses and verified that gilteritinib, ipatasertib, prinomastat, and marimastat have high antiviral activity [10]. Multi‐omics analysis of silicosis revealed that phosphorylated epidermal growth factor receptor (p‐EGFR) and SYK (p‐SYK) are potential therapeutic targets. The findings were validated as it was demonstrated that fostamatinib and gefitinib effectively improve lung dysfunction and inhibit the progression of inflammation and fibrosis [11].

Although most clinical fatalities associated with EV‐A71 arise from neurological complications, skeletal muscle in mouse models is considered a critical initial site of viral replication and a potential gateway to the central nervous system (CNS). Studies on several neurotropic viruses (including classical rabies virus) have established a similar dissemination pattern: the pathogen first amplifies in skeletal muscle, then invades motor neuron terminals at the neuromuscular junction, and is subsequently transported retrogradely through the spinal cord to the brainstem [12]. In this study, we observed the highest viral loads and the most severe pathological damage in skeletal muscle, characterized by necrotizing myositis, whereas neural injury was comparatively mild. Systematic analysis of host responses in skeletal muscle, particularly antiviral and inflammatory pathways, may reveal potential strategies to block viral dissemination to the CNS. Our studies using EV‐A71 animal models have shown that viral replication and tissue pathology follow distinct temporal patterns. Specifically, 2 days post infection (dpi) represents the peak of viral replication, whereas 5 dpi corresponds to the peak of pathological injury, making these time points critical for dissecting host‐virus interactions. Thus, selecting 2 and 5 dpi for proteomic analysis allows us to capture both the viral replication phase and the host injury response, providing complementary insights into EV‐A71 pathogenesis.

To investigate host responses during EV‐A71 infection, we performed comprehensive transcriptomic, proteomic, and phosphoproteomic analyses of skeletal muscle from infected BALB/c mice at two distinct time points, representing early and late stages of infection. We quantitatively assessed global changes in gene expression, protein abundance, and phosphorylation dynamics in response to viral infection. This multi‐omics dataset elucidates EV‐A71‐induced alterations in the target tissue and integrates omic‐based target discovery with drug screening and functional validation, providing mechanistic insights and guiding potential therapeutic strategies.

## 2 Results

### 2.1 Multi‐omics Profiling of Muscle Tissue From Control and EV‐A71‐Infected Mice

To investigate the pathogenic mechanism of the EV‐A71 virus, a multi‐omics analysis was performed using BALB/c mice. Twelve 7‐day‐old BALB/c mice were intraperitoneally (i.p.) injected with PBS as a control, and hindlimb skeletal muscles were harvested at 0, 2, and 5 days. Eight BALB/c mice were i.p. infected with the EV‐A71, and hindlimb skeletal muscle samples were collected for viral load determination and pathological analysis at 2 and 5 dpi. Moreover, we performed a time‐resolved multi‐omics analysis to examine the effects of viral infection on mRNA expression, protein abundance, and phosphorylation levels.

H&E staining showed more pronounced skeletal muscle necrosis and inflammatory cell infiltration at 5 dpi compared with earlier time points (Figure S1A–E). Viral replication in muscle tissue was assessed at each time point, showing significantly higher viral loads at 5 dpi than at 2 dpi, consistent with previous studies [13] (Figure S1F). Principal component analysis (PCA) was used to analyze the quality of the transcriptomic, proteomic, and phosphoproteomic data. The results showed tight clustering of samples within each group, indicating high quantitative reproducibility between biological replicates. The EV‐A71 virus infection group (2 dpi) exhibited substantial overlap with the control group, whereas the EV‐A71 infection group (5 dpi) was the most common outlier, indicating that EV‐A71 infection induced progressive molecular changes in mice over time (Figure S2A–C). For basic bioinformatic analysis, data from each time point of the virus‐infected group were compared with their respective mock‐infected controls. We defined a gene, protein, or phosphosite as significantly differentially expressed postinfection on the basis of a Student's *t*‐test *p* value <0.05 and fold change (FC) >2 or <0.5 at any time point. A total of 14,385, 8638, and 4416 quantifiable genes, proteins, and phosphoproteins, respectively, were observed (Figure S2D–F, Table S1). A Venn diagram was constructed to compare differentially expressed genes, proteins, and phosphosites across the two time points postinfection, indicating that some molecules were continuously altered throughout infection, whereas the majority of molecules were altered at the late stage (Figure S2G–I). In addition, 281 molecules exhibited significant changes across all three omics analyses at 5 dpi, whereas the molecular variation at 2 dpi was quite different (Figure S2J–K). Together, these findings demonstrate that EV‐A71 infection leads to severe pathological damage accompanied by extensive molecular alterations.

### 2.2 Temporal Transcriptome and Proteomic Analysis of EV‐A71‐Infected Mice

**FIGURE 1:**
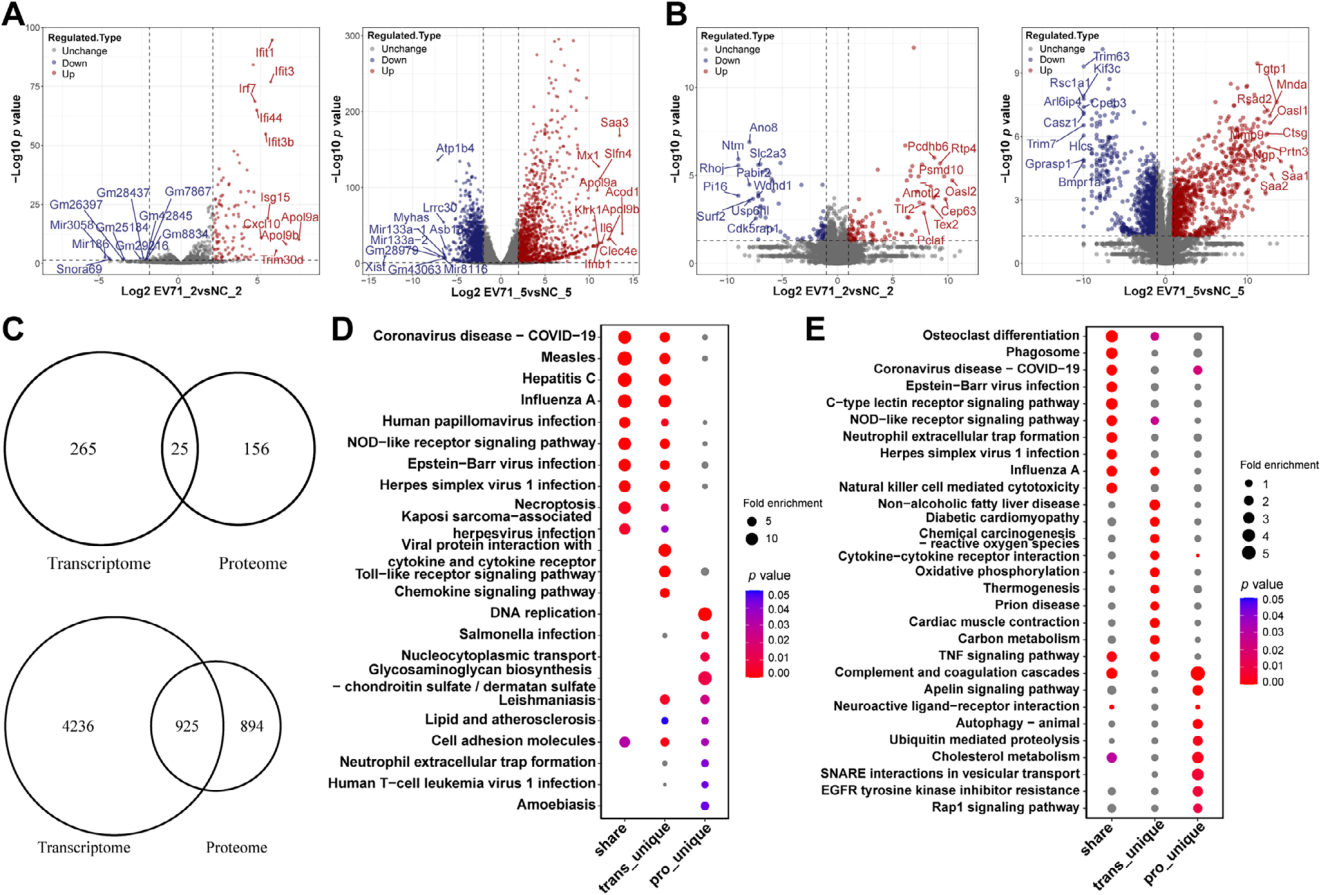
Basic and functional analyses of the transcriptome and proteome. (A and B) Volcano plot. The upregulated molecules are shown in red, the downregulated molecules are shown in blue, and the molecules with no significant difference are shown in gray. (A) Volcano plot showing differential gene expression. (B) Volcano plot showing differential protein abundance. (C) Venn diagram of the transcriptome and proteome. The left circle represents the differentially expressed transcripts, and the right circle represents the DEPs. The left picture shows the results at 2 dpi, and the right picture shows those at 5 dpi. (D and E) Functional enrichment analyses for shared and unique molecules in the transcriptome and proteome at 2 dpi (D) and 5 dpi (E). The top 10 KEGG pathways enriched in each group are integrated and presented in the bubble plots.

As described above, the number of significantly dysregulated genes and proteins increased over the course of infection. Transcriptome analysis revealed that EV‐A71 upregulates interferon‐inducible genes (*Ifit1*, *Ifit3*, *Ifit3b, Apol9a*, *Apol9b*, *Ifnb1*, and *Mx1*) [14, 15, 16] and activates a proinflammatory signature (*Cxcl10* and *Il6*). Interestingly, the anti‐inflammatory genes *Acod1* were markedly upregulated at 5 dpi, likely reflecting a compensatory feedback mechanism to curb localized inflammation. By driving itaconate biosynthesis, *Acod1* attenuates excessive pro‐inflammatory cytokine release, thus fine‐tuning the balance between antiviral immunity and tissue integrity and preventing irreversible muscle injury induced by hyperinflammatory cascades [17, 18] (Figure 1A). Proteomic data showed that the proteins significantly altered at the two time points were largely distinct (Figure 1B). To validate these findings, we selected four of the most significantly upregulated and downregulated genes and three proteins from the top 10 most altered molecules for qPCR and Western blot (WB). The results confirmed significant changes in these genes and proteins, consistent with the multi‐omics data (Figure S3A–C).

A Venn diagram revealed a progressive increase in overlap between transcriptome and proteome changes, with 25 and 925 common molecules at 2 and 5 dpi, respectively (Figure 1C). KEGG analyses were conducted on shared and unique molecules (Figure 1D,E). At 2 dpi, shared and transcriptome‐specific molecules were primarily enriched in viral infection and innate immunity‐related pathways, whereas proteome‐specific proteins were enriched in DNA replication, nucleoplasmic transport, and cell adhesion molecules. By 5 dpi, pathway enrichments diverged further: common molecules retained enrichment in viral and immune pathways, transcriptome‐specific genes were associated with core cellular functions such as oxidative phosphorylation, thermogenesis, myocardial contraction, and carbon metabolism, and proteome‐specific proteins were enriched in pathways related to intra‐ and extracellular signaling and regulation (apelin signaling pathway, neuroactive ligand‐receptor interaction, and Rap1 signaling pathway). Interestingly, proteins associated with the neutrophil extracellular trap formation (NETs) pathway were altered at 2 dpi, whereas co‐alteration of genes and proteins was found at 5 dpi.

**FIGURE 2:**
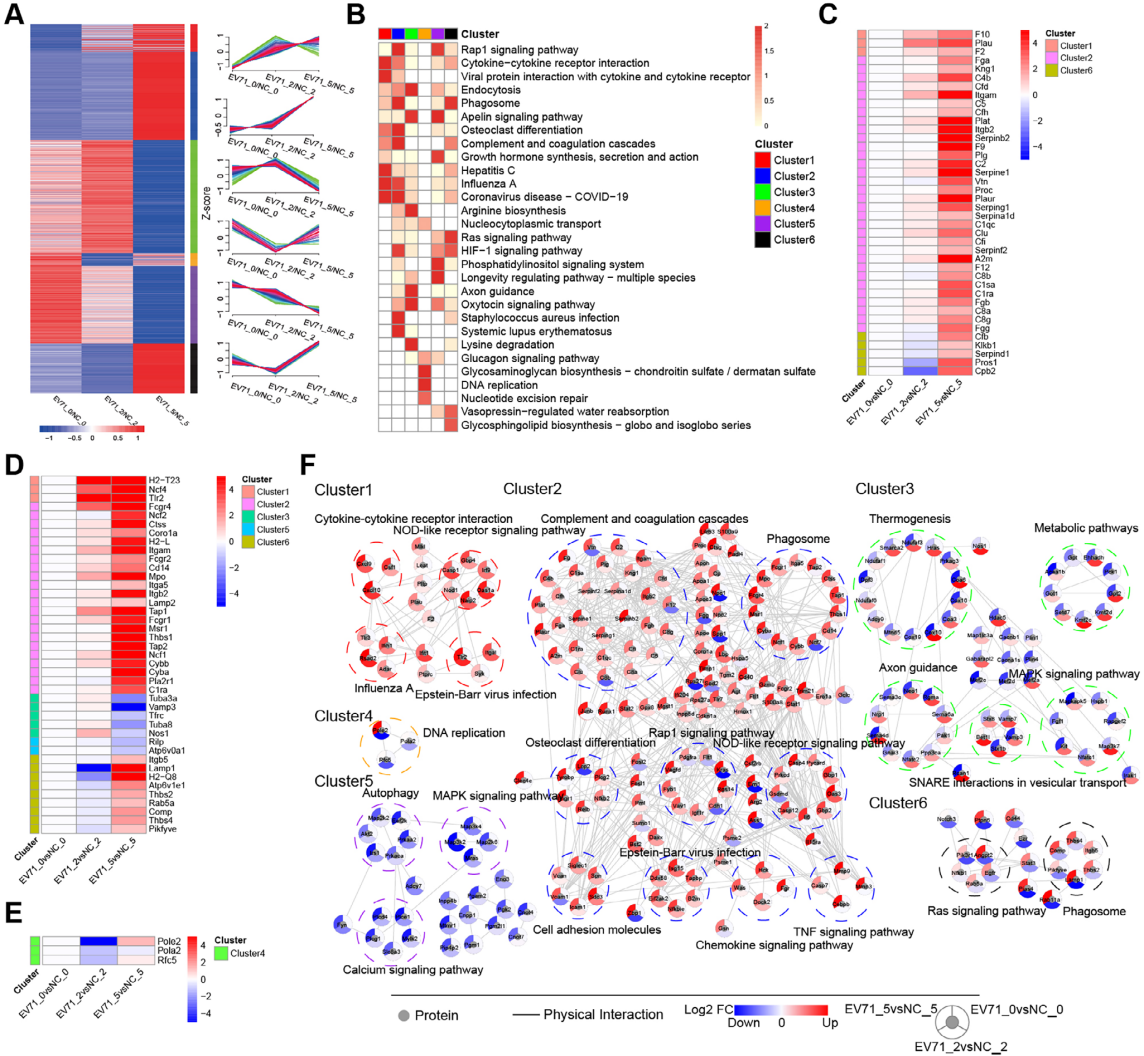
Characterization of temporal changes in proteome after EV‐A71 infection. (A) Clusters depicting protein abundance at 0, 2, and 5 dpi. The DEPs at each time point were integrated into a union set. On the basis of the protein FC at each time point, fuzzy *C*‐means were used to separate the DEPs into six clusters. (B) Heatmap of KEGG functional enrichment analysis results for each cluster in (D). Each cluster represents the top 5 pathways, and the color of the square represents the degree of enrichment (*p* value). (C–E) The differential expression of enriched proteins in the complement and coagulation cascades (C), phagosome (D), and DNA replication (E) pathway. (F) PPI network of proteins in the enriched KEGG pathways in the cluster shown in (A). The red‐to‐blue color gradient represents the log2 FC of the corresponding proteins at each time point. EV‐A71, enterovirus A71.

To determine the temporal specificity of the differences in expression after EV‐A71 infection, we sorted DEPs from each time point and converted them into a union set. We then used the fuzzy *C*‐means algorithm and separated the FCs of the molecules at the two time points into six clusters (the data on Day 0 were set to one), reflecting significant functional enrichment (Figure 2A–E). The numbers of DEPs in each cluster were 145, 465, 597, 69, 408, and 263. Moreover, protein–protein interaction (PPI) analysis was used to explore the specific expression patterns of the proteins in each cluster (Figure 2F).

Cluster 1 comprised proteins that were continuously upregulated following viral infection and were mainly enriched in viral infection‐related pathways, including influenza A and Epstein–Barr virus infection, as well as inflammation‐ and immune‐related pathways. Cluster 2 included proteins upregulated in the late stage of viral infection, mainly associated with innate immune response. Cluster 6 consisted of proteins slightly downregulated early but significantly upregulated at late stage, enriched in phagocytosis‐ and cell proliferation‐related pathways. Within the complement and coagulation cascades and phagosome pathway, distinct proteins showed different temporal trends, suggesting phase‐specific immune roles. In the phagosome pathway, Ncf4, Tlr2, and H2‐T23 were continuously upregulated; 22 proteins, such as Fcgr4 and Ncf2, were upregulated in the late stage of infection; and nine proteins, such as Itgb5 and Lamp1, were downregulated early and upregulated later. In the complement and coagulation cascades, Clusters 1, 2, and 6 included 3, 32, and 5 proteins, respectively. Most proteins in Cluster 3 were upregulated early and downregulated later, and their functions were enriched for thermogenesis, metabolic pathways, and axon guidance. Proteins within Cluster 4 exhibited a dynamic response to infection and were enriched in DNA replication (Pole2, Pola2, and Rfc5). In addition, some proteins associated with autophagy, the MAPK signaling pathway, and the calcium signaling pathway were consistently downregulated after EV‐A71 infection (Cluster 5). Integrated transcriptomic and proteomic analyses uncovered overlapping and unique molecular changes, as well as stage‐specific functional responses to EV‐A71 infection.

### 2.3 EV‐A71 Infection‐Induced NETs

**FIGURE 3:**
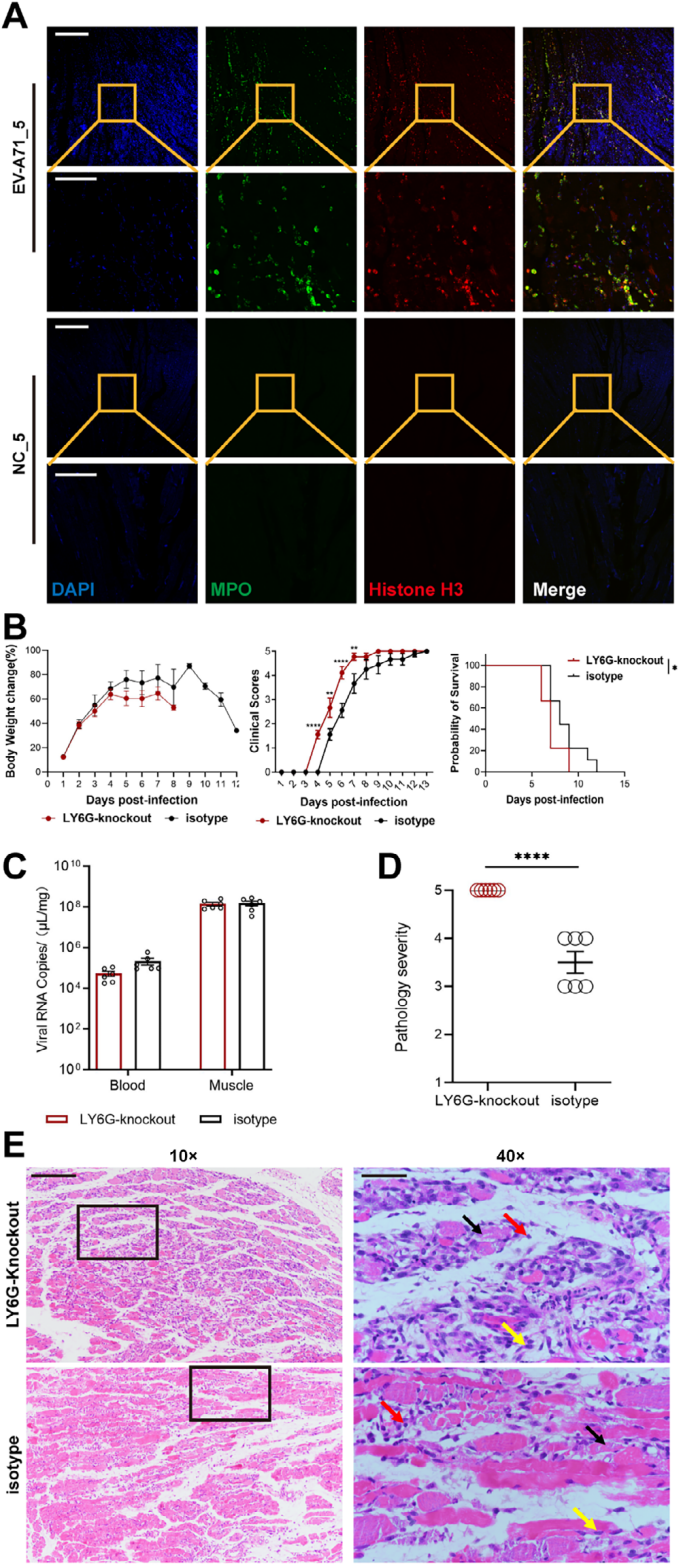
EV‐A71 virus infection triggers NETs. (A) Immunofluorescence analysis in skeletal muscle. Skeletal muscle sections from EV‐A71‐infected and NC mice at Day 5 were stained with an anti‐MPO antibody (green), an anti‐histone H3 antibody (red), and DAPI (blue). The image was captured using 10× (bar = 250 µm) and 40× (bar = 75 µm). Seven‐day‐old neonatal BALB/c mice were intraperitoneally injected with 100 µL of EV‐A71 per mouse. Mice in the LY6G‐Knockout group were administered 100 µL of anti‐LY6G (10 mg/kg) on alternate days, whereas mice in the isotype group were given rat IgG2a isotype control (10 mg/kg) on alternate days. Both groups were observed daily (*n* = 8) and dissected and sampled for blood and muscle at 5 dpi (*n* = 6). (B) The body weights changes, clinical scores, and survival rates were recorded from 1 to 14 dpi. (C) The viral loads in blood and muscle. (D) Pathological grading in LY6G‐knockout and isotype groups. (E) Skeletal muscle sections from LY6G‐knockout and isotype groups were stained with H&E. Black arrows represent muscle fiber necrosis; yellow arrows represent connective tissue hyperplasia; red arrows represent inflammatory cell infiltration. The image was captured using 10× (bar = 200 µm) and 40× (bar = 50 µm), black box for 40× field of view. Statistical analysis of survival rates using the log‐rank test and body weight was performed among the two groups using Mann‒Whitney *U* tests, *p < 0.05. All data are represented as the mean ± SEM, and *t*‐test and one‐way ANOVA were used for statistical analysis: *p < 0.05, **p < 0.01, ***p < 0.001, ****p < 0.0001.

Transcriptomic and proteomic analyses revealed that the NETs pathway was activated following viral infection. NETs are network structures composed of neutrophil granule proteins, DNA, and chromatin that can recognize and capture bacteria, viruses, or fungi. To explore the potential regulatory role of host NETs during viral infection, we performed a preliminary validation. Immunofluorescence analysis revealed extensive neutrophil accumulation and high NETs expression following infection (Figure 3A). Neutrophil knockout accelerated hindlimb paralysis and led to earlier mortality in mice (Figure 3B). Although no significant difference was found in viral load (Figures S4A and S5; Figure 3C), the pathological manifestations were more severe, with massive necrosis of muscle cells and concomitant presence of infiltration of inflammatory cells other than neutrophils (Figure 3D,E). These findings suggest that NETs may play a protective role in mitigating tissue damage during EV‐A71 infection.

### 2.4 EV‐A71 Infection Leads to Protein Phosphorylation and Regulates Host Kinase Signaling

**FIGURE 4:**
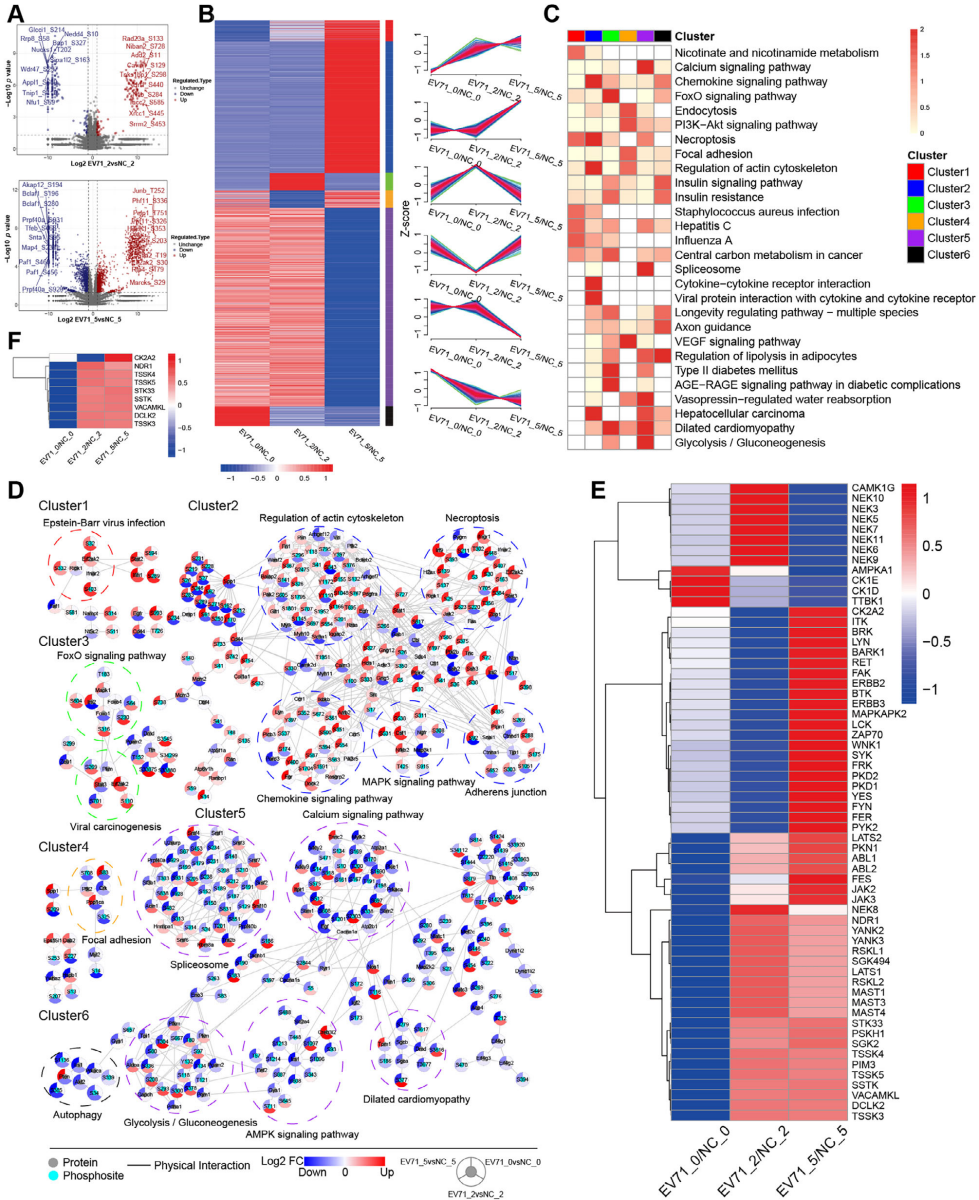
Phosphoproteomic profiling revealing dysregulated pathways and kinases after EV‐A71 infection. (A) Volcano plot showing the activation and inhibition of phosphorylation sites. (B) Differentially expressed phosphosites at each time point were integrated into a union set. On the basis of the phosphosite FC at each time point, fuzzy *C*‐means was used to separate the differentially expressed phosphosites into six clusters. (C) Heatmap of KEGG functional enrichment analysis results for each cluster in (B). Each cluster represents the top 5 pathways, and the color of the square represents the degree of enrichment (*p* value). (D) PPI network of phosphosites and proteins in the enriched top 5 KEGG pathways in the cluster shown in B. The red‐to‐blue color gradient represents the log2 FC of the corresponding proteins at each time point. Heatmap of predicted kinases that may have had significantly altered activity after EV‐A71 infection; intersection (E) and union (F) of two time points. Kinase activity is represented by NES values. EV‐A71, enterovirus A71.

Consistent with the proteomic results, the number of differentially expressed phosphorylation sites was significantly greater during the late stage of infection. In particular, we observed a significant increase in the phosphorylation of proteins related to DNA repair, including Rap23a (S133, 2 dpi) [19], Xrcc1 (S445, 2 dpi) [20], Phf11 (S336, S326, 5 dpi) [21], and Pelp1 (T751, 5 dpi) [22]. Proteins associated with cell growth, including Srrm2 (S453, 2 dpi) [23], Tacc2 (S585, 2 dpi) [24], and Junb (T252, 5 dpi), also exhibited enhanced phosphorylation. Additionally, we observed increased phosphorylation of Rtp4, a protein previously identified as an effective IFN‐inducing inhibitor of the replication of SARS‐CoV‐2, yellow fever virus, and dengue virus [25, 26]. The potential role of Rtp4 in the treatment of the EV‐A71 virus warrants further investigation. By contrast, phosphorylation levels of Paf1 and Bclaf1, two key transcriptional and posttranscriptional regulators involved in antiviral immunity, were significantly downregulated, suggesting that EV‐A71 may exploit perturbations of these regulators to manipulate host transcriptional programs and facilitate immune evasion (Figure 4A). To study the effects of virus infection on host protein phosphorylation, we separated the FCs of the differentially expressed phosphosites at the three time points into six clusters (Figure 4B). Functional and pathway enrichment analyses were then performed for each cluster (Figure 4C,D).

Clusters 1 and 2 included phosphorylation sites that were upregulated during EV‐A71 infection. Cluster 1 contained phosphosites that were consistently upregulated and enriched in pathways related to necroptosis, metabolism (pyrimidine metabolism and nicotinamide and nicotinamide metabolism‐related pathways), and viral infection‐related pathways (influenza A and hepatitis C). Cluster 2 sites tended to be upregulated at the late stage of infection and were associated with cytokine‒cytokine receptor interactions, chemokine signaling pathways, and viral protein interactions with cytokines and cytokine receptors. Clusters 3 and 4 represented phosphorylation sites that changed at 2 dpi. In the early stages of infection, hyperphosphorylated proteins were enriched in pathways linked to type II diabetes mellitus, the FoxO signaling pathway, and dilated cardiomyopathy, whereas hypophosphorylated proteins were associated with the VEGF signaling pathway, endocytosis, focal adhesion, and the PI3K/Akt signaling pathway. Clusters 5 and 6 comprised phosphorylation sites that were downregulated during infection. Cluster 5 included sites whose phosphorylation decreased at the late stage and were enriched in pathways such as spliceosomes, glycolysis and gluconeogenesis, the calcium signaling pathway, and dilated cardiomyopathy. The phosphorylation sites in Cluster 6 were consistently downregulated and enriched for axon guidance, insulin resistance, and insulin signaling pathways, among others.

Protein phosphorylation is an important PTM catalyzed by protein kinases. We predicted alterations in kinase activity based on the modulation of known substrates to obtain additional information. Quantifiable phosphosites were submitted to iGPS to determine the relationships between phosphorylated proteins and kinases. The GSEA algorithm was used to predict normalized enrichment score (NES). Kinases filtered by GSEA *p* < 0.05 and NES >1 (activated) or <−1 (inhibited) were defined as having significantly altered activity. Phosphoproteomic enrichment analysis revealed 62 predicted alterations in kinase activity across all time points. At 2 dpi, 33 kinases were activated and 16 inhibited, whereas at 5 dpi, 49 were activated and four inhibited. Nine kinases showed consistent changes at both stages, among which CK2A2 shifted from inhibition early to activation later, whereas the others remained activated (Figure 4E,F). Phosphoproteomic analysis revealed that EV‐A71 infection induces pronounced and time‐dependent changes in host kinase activity.

### 2.5 KEGG Enrichment Analyses of the Transcriptome, Proteome, and Phosphoproteome

We integrated the transcriptome, proteomic, and phosphoproteomic datasets using KEGG analysis to explore the main pathways affected by EV‐A71 infection (Figure S6 and Table S2). Consistent functional enrichments were observed across the three omics levels. The expression levels of genes and proteins and phosphorylation sites related to immune pathways, including NOD‐like receptor signaling pathway, phagosome, cytokine‒cytokine receptor interaction, and complement and coagulation cascades, were significantly upregulated after infection, whereas the expression of molecules related to cellular regulatory pathways such as thermogenesis, the spliceosome, and oxidative phosphorylation was downregulated. Moreover, we also found that molecules of disease‐related pathways showed altered expression after EV‐A71 infection. Molecules associated with viral infection pathways (influenza A, hepatitis C, and Epstein–Barr virus infection) were upregulated, and neurological molecules such as those related to Alzheimer's disease and Parkinson's disease were downregulated. At the protein level, components of the neutrophil extracellular trap pathway were significantly upregulated. In addition, metabolic pathways displayed both up‐ and downregulated gene expression patterns. Overall, the integrated multi‐omics data reveal that EV‐A71 infection induces robust immune responses accompanied by broad metabolic and disease‐related pathway alterations.

### 2.6 Mapping Kinase Activities to Drugs and Testing EV‐A71 Therapies

**FIGURE 5:**
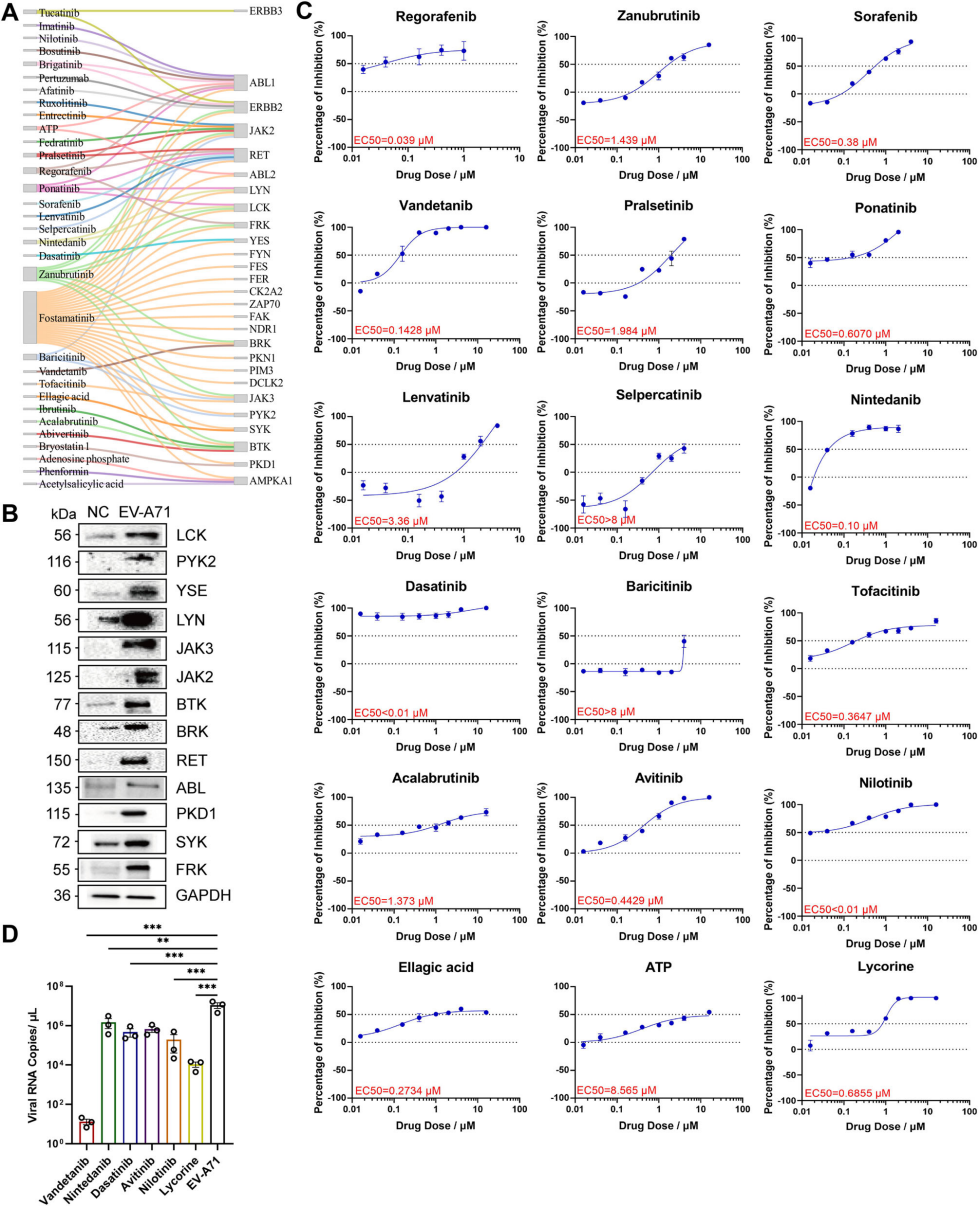
Mapping regulated kinases to kinase inhibitors identifies EV‐A71 therapies. (A) Drugs (left) mapped to kinases (right) whose activity was regulated by EV‐A71 infection. Lines connecting them indicate known kinase targets for each drug. (B) Western blotting images showing kinase activation in control (left) and EV‐A71‐infected (right) mice. (C) RD cells were pretreated with drugs at the indicated doses and then infected with EV‐A71 for 72 h. The viral load in the cell supernatants was determined. The inhibitory effect of drug treatment on EV‐A71 replication. The graphs show the results for regorafenib, zanubrutinib, sorafenib, vandetanib, pralsetinib, ponatinib, lenvatinib, selpercatinib, nintedanib, dasatinib, baricitinib, tofacitinib, acalabrutinib, avitinib, nilotinib, ellagic acid, ATP, and lycorine (*n* = 3). (D) Viral load in the cell supernatants of the vandetanib (4 µM), nintedanib (4 µM), dasatinib (4 µM), avitinib (8 µM), nilotinib (8 µM), and lycorine (8 µM) treatment groups and the control group. All data are represented as the mean ± SEM, and one‐way ANOVA was used for statistical analysis. EV‐A71, enterovirus A71. *p < 0.05, **p < 0.01, ***p < 0.001.

Multi‐omics analyses indicated that the molecular changes were most significant at the late stage of infection. To identify potential therapies, we mapped drugs to the most differentially regulated kinase activities at 5 dpi (Figure 5A and Table S3). A total of 32 drugs were mapped to 27 kinases, with some drugs targeting multiple kinases. Of these, fostatinib demonstrated low specificity, whereas AMPKA1 was inhibited following viral infection. Consequently, the 16 kinases were validated. WB analysis revealed that 14 kinases (Lck, Brk, Yse, Lyn, Jak3, Jak2, Btk, Brk, Ret, Abl1/2, Pkd1, Frk, and Syk) were activated following virus infection (Figure 5B). Subsequently, a total of 17 drugs were selected for further testing (selection of 2–3 corresponding drugs for each kinase), with lycorine acting as a positive control. Here, the antiviral efficacy (via qRT‐PCR) and cellular toxicity (via MTS) of 18 total drugs were tested in the RD cell line (Figure 5C and Figure S7A–R).

**FIGURE 6:**
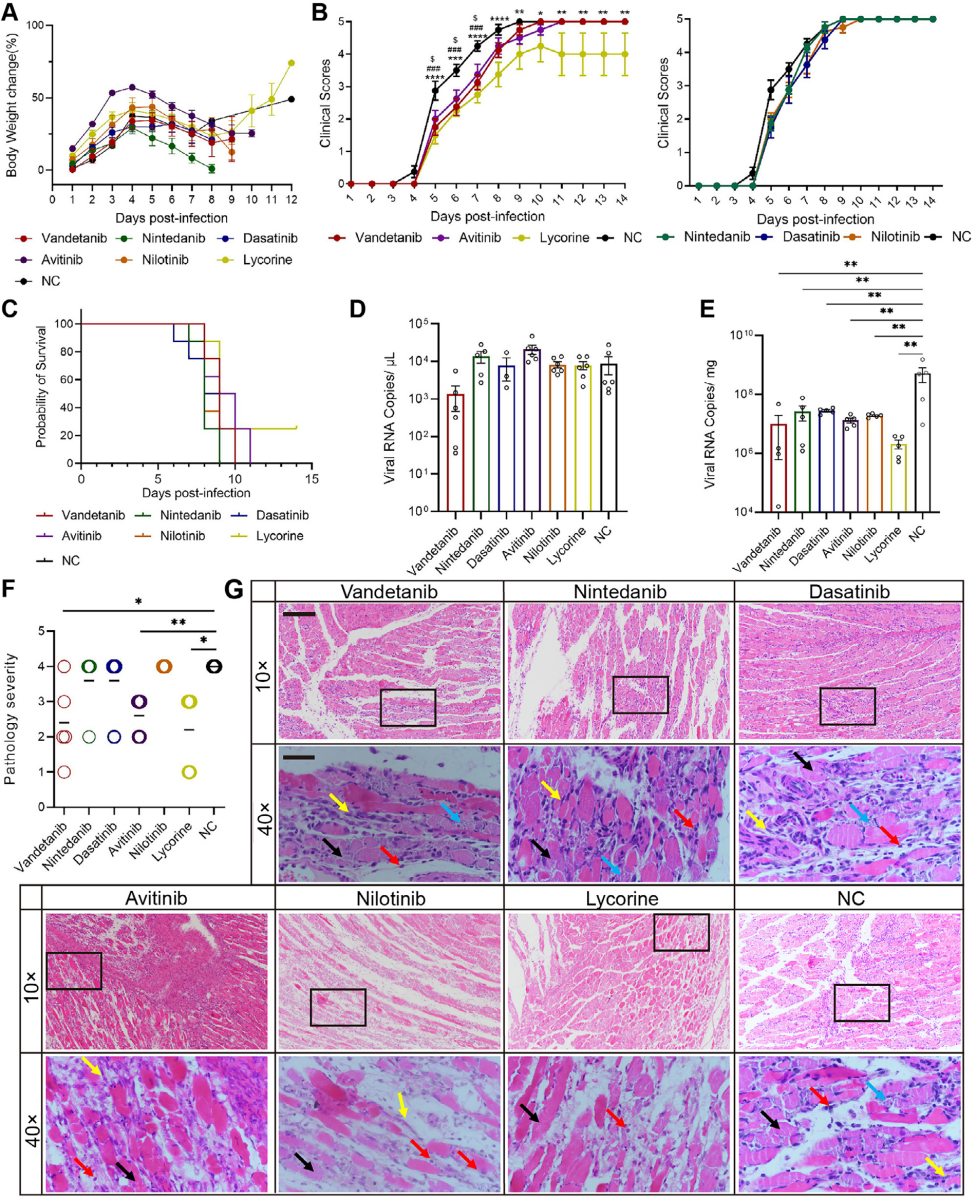
Kinase inhibitor drug inhibited viral replication in mice. Ten‐day‐old BALB/c mice were infected with 100 LD_50_ or 0.1 LD_50_ EV‐A71 virus by intraperitoneal injection. Vandetanib (10 mg/kg/day), nintedanib (5 mg/kg/day), dasatinib (10 mg/kg/day) and avitinib (5 mg/kg/day) were administered by gavage, and nilotinib (5 mg/kg/day) and lycorine (0.6 mg/kg/day) were administered intraperitoneally for 2 h prior to infection and then daily. PBS was used as treatment in the NC group. Both groups were observed daily (100 LD_50_, *n* = 8) and dissected and sampled for blood and muscle at 5 dpi (0.1 LD_50_, *n* = 6). The body weights changes (A), clinical scores (B), and survival rates (C) were recorded from 1 to 12 dpi. Blood (D) and muscle (E) samples were collected to detect the viral loads at 5 dpi. (F and G) Skeletal muscle sections from vandetanib‐, nintedanib‐, dasatinib‐, avitinib‐, nilotinib‐, lycorine‐, and PBS‐treated mice were stained with H&E. Black arrows represent muscle fiber necrosis; Blue arrow nucleolysis, rare calcification; yellow arrows represent connective tissue hyperplasia; red arrows represent inflammatory cell infiltration. The image was captured using 10× (bar = 200 µm) and 40× (bar = 50 µm), black box for 40× field of view. All data are represented as the mean ± SEM. One‐way ANOVA and two‐way ANOVA were used for statistical analysis: *($) *p* < 0.05, **p < 0.01, ***(###) *p* < 0.001, ****p < 0.0001. * (lycorine vs. NC), $ (avitinib vs. NC), # (vandetanib vs. NC) in (B).

All 17 tested drugs inhibited EV‐A71 infection in vitro. Quantitative PCR analysis indicated that vandetanib, nintedanib, dasatinib, avitinib, nilotinib, and lycorine, which are intracellular inhibitors of tyrosine kinases, had stronger antiviral effects against EV‐A71. On the basis of their antiviral efficacy in vitro, six drugs showing the most promising effects were selected for in vivo validation. The viral loads in the six drug treatment groups and the control group were 9.73E + 00 copies/µL (4 µM), 1.51E + 06 copies/µL (4 µM), 4.81E + 05 copies/µL (4 µM), 6.81E + 05 copies/µL (8 µM), 1.94E + 05 copies/µL (8 µM), 1.09E + 04 copies/µL (8 µM), and 4.02E + 06 copies/µL, respectively (Figure 5D). Considering the toxicity of the various drugs, we tested the efficacy of the drugs in 10‐day‐old BALB/c mice (Figure S4B). Clinical observations revealed a consistent trend in the body weights of the mice, with an initial increase followed by a subsequent decrease (Figure 6A). Clinical scores indicated that vandetanib and avitinib could delay the onset of paralysis in the mice yet failed to enhance their survival rate, and the group receiving the positive drug lycorine exhibited a 25% survival rate, with the symptoms being delayed (Figure 6B,C). Vandetanib inhibited BRK, a regulator of the EGFR/HER2 pathway, whereas avitinib targeted BTK, a key molecule in BCR signaling (Figure S7S). However, we found that each of the six drugs reduced the viral load in muscle (Figure 6D) but not in blood (Figure 6E) of mice challenged with sublethal doses of virus. The viral load in the muscles decreased from 5.29E + 08 to 1.20E + 05, 2.87E + 05, and 2.79E + 05, 1.32E + 07, 1.92E + 07, 2.10E + 06 copies/mg, respectively, at 5 dpi. Histological analysis of muscle tissue from mice revealed the presence of myofibrillar necrosis, foci of calcification, connective tissue hyperplasia, and scattered lymphocytic and granulocytic infiltration. Treatment with vandetanib, avitinib, and lycorine reduced necrosis and inflammatory cell infiltration in muscle (Figure 6F,G). Kinase inhibitors blocked viral replication in vitro but failed to improve survival in EV‐A71–infected mice, suggesting limited in vivo efficacy.

## 3 Discussion

Multi‐omics analyses provide the unique advantage of capturing molecular changes across multiple regulatory layers, offering a comprehensive view of host responses during EV‐A71 infection. Previous studies on EV‐A71 have mainly relied on two‐dimensional cell models to investigate virus–host interactions and identify antiviral targets. More recently, hiPSC‐derived skin organoids have been used to examine the molecular mechanisms and pathological changes at different time points after EV‐A71 infection, leading to the identification of HMGB1 as a key host factor [27]. However, both approaches have inherent limitations and cannot fully recapitulate viral replication in vivo. In this study, we for the first time employed a Balb/c mouse model to conduct multi‐omics analyses of EV‐A71 infection, systematically characterizing the dynamic changes in gene expression, protein abundance, and phosphorylation during the course of infection. Furthermore, using the multi‐omics data to identify key kinase targets, we screened potential therapeutic drugs and validated both their antiviral effects and the functional roles of the kinases. By integrating multi‐omics, target discovery, drug screening, and target validation, our study provides a comprehensive framework linking molecular mechanisms to therapeutic intervention.

In this study, we observed temporal change in protein expression over time after EV‐A71 infection. The results showed that the continuously upregulated proteins were involved in a variety of virus infection‐related pathways, such as influenza A, coronavirus disease‐COVID‐19 and hepatitis C. Meanwhile, EV‐A71 infection induces persistent activation of immune pathways, including phagosome, complement, and coagulation cascades. Viral invasion triggers one or more complement pathways (lectin, classical, or alternative) to recognize pathogens, as observed in human respiratory syncytial virus infections [28]. Phagosomes critically mediate microbial clearance and antigen presentation. Pathological analyses revealed severe late‐stage inflammatory cytokine storms in infected mice. We speculate that early‐phase immune activation functions to contain viral dissemination through antiviral effector mechanisms. In contrast, late‐phase immune hyperactivation, together with disruptions in coagulation homeostasis, contributes to muscle damage. This biphasic response likely represents the central mechanism underlying the pathological manifestations observed in the murine model. Striking an appropriate balance between these phases could be a critical focus for future research on host defense against EV‐A71 infection. However, the KEGG pathways associated with the continuous downregulation of proteins, such as the growth hormone synthesis, secretion, and action pathway and the Rap1 signaling pathway, are mostly related to cell growth. Researchers observed a marked downregulation of growth hormone in ZIKV‑infected BALB/c suckling mice, which, via the hypothalamic–pituitary–thyroid axis, induced irreversible growth retardation and impairments in learning and memory [29]. Inhibition of the Rap1 signaling pathway could impair cell proliferation, differentiation, and cytoskeletal remodeling, thereby disrupting tissue homeostasis, whereas downregulation of growth hormone‐related pathways may reduce metabolic activity and regenerative capacity, creating a favorable environment for viral replication. Collectively, suppression of these growth‐ and signaling‐related pathways may weaken host defenses, disturb tissue and metabolic balance, and ultimately exacerbate EV‐A71 pathogenesis.

Neutrophil cells are the first line of defense of the host against infection and injury, and their mechanisms of action include phagocytosis and degranulation. However, when neutrophils encounter microorganisms that are too large for phagocytosis or pathogens that escape phagosome destruction by escaping into the cytoplasm, another defense pathway, that of NETs, is initiated [30]. NETs have been observed in various viral diseases and represent a “double‐edged sword” in host defense. On one hand, excessive NETs can trigger uncontrolled inflammatory responses, accompanied by the release of mediators such as ROS and MPO, leading to immune overactivation and tissue damage. For instance, NETs exacerbate liver injury in fulminant viral hepatitis (FVH) by promoting fibrin deposition and inflammation, whereas in SARS‐CoV‐2 infection, neutrophil accumulation and NET release have been linked to severe inflammatory pathology [31, 32]. On the other hand, NETs act as antimicrobial weapons that can restrict and eliminate pathogens, thereby contributing to antiviral immunity. Recent studies demonstrated that the Piezo1 ion channel drives NETs and regulates macrophage differentiation during influenza virus infection [33]. Previous studies have shown that the VP1 protein of EV‐A71 can directly induce pulmonary NET formation in a peptidylarginine deiminase 4 (PAD4)‐dependent manner, leading to pulmonary edema [34]. However, these studies were limited in that they only employed the VP1 protein rather than live viral infection, and the mechanisms and functional consequences of NET formation were not fully elucidated. Integrated multi‐omics analyses revealed the rapid activation of the NETs pathway at the protein level, suggesting a “pre‐armed defense” strategy: neutrophils promptly release pre‐synthesized antimicrobial proteins (MPO) via degranulation to intercept viruses, whereas the upregulation of multiple histone H3 (H3c7, H3c13, H3c14, H3c3, H3c15, H3c6, H3c2, H3c4) promotes chromatin decondensation, forming the core scaffold of extracellular traps. Histone H3 and its posttranslationally modified isoforms, such as citrullinated H3 (CitH3), are known to directly engage in antimicrobial defense and inflammatory modulation. This citrullination process, mediated by the PAD4 enzyme, plays a critical regulatory role in the early phase of NETs [35]. Importantly, these early‐phase changes in NET‐associated proteins precede transcriptional reprogramming, indicating that interventions targeting post‐translational modifications (e.g., PAD4‐catalyzed citrullination) may offer superior timeliness compared to transcriptional regulation. Immunofluorescence analyses further confirmed that EV‐A71 virus infection resulted in elevated NETs expression in mouse muscle. Functional depletion of neutrophils in vivo significantly exacerbated disease progression, accelerating mortality and heightening muscular pathology. This phenotypic aggravation suggests neutrophils play a crucial cytoprotective role in viral pathogenesis, potentially through modulating inflammatory cascades and containment of local infection. Depletion of neutrophils did not affect viral loads within infected muscle of mice, implying that neutrophils primarily function as orchestrators of immune‐mediated tissue preservation rather than as direct mediators of viral clearance. NETs appear to play a beneficial role during EV‐A71 infection, potentially modulating inflammatory responses and influencing disease outcomes. In a CVA6‐induced myocarditis model, neutrophil depletion has been reported to attenuate cardiac inflammation and tissue injury [36]. This reveals that the function of neutrophils is not universal but is highly dependent on varying of the specific virus and the infected organ. Nevertheless, their precise mechanisms of action remain to be further elucidated. A limitation of our study is that we employed Ly6G neutralizing antibody treatment to deplete neutrophils, rather than utilizing Ly6G knockout mice. Although antibody‐mediated depletion is a widely used approach and we confirmed its effectiveness by flow cytometry, genetic knockout models are generally considered more robust and may provide more definitive evidence. Future studies employing Ly6G‐deficient mice and more specific NET inhibitors may provide clearer insights into the roles of neutrophils and NETs during EV‐A71 infection.

Protein phosphorylation is a key PTM that regulates protein function across nearly all cellular processes. Phosphoproteomics has improved our understanding of the function of kinases by revealing new downstream substrates and revealing their biological functions [37]. As phosphorylation changes reflect kinase activity, we profiled kinase activities on the basis of changes in their annotated substrates and identified 53 altered kinases at 5 dpi. The CK1 family comprises serine/threonine‐protein kinases that regulate membrane transport, cell division, DNA repair, the circadian rhythm, and nuclear localization [38, 39]. Kinase activity profiling analysis revealed that Ck1d and Ck1e activities are significantly reduced by EV‐A71 infection, providing further evidence to support the hypothesis that viral infection can inhibit cell growth. Conversely, several kinases related to immune regulation were activated. For example, Lck may promote the development, activity, and proliferation of T‐cells [40]; Btk may regulate the development, maturation, and differentiation of immature B‐cells as well as the proliferation and survival of B‐cells [41]; and Lyn plays an important role in the initiation of proinflammatory and inhibitory signaling pathways in bone marrow immune cells (neutrophils, dendritic cells, monocytes, macrophages) and B lymphocytes [42].

Kinases represent ideal drug targets. In this study, WB analysis revealed upregulation of 14 kinases following viral infection. Accordingly, 2–3 corresponding inhibitors per kinase (17 in total) were selected for antiviral assessment, using lycorine as a positive control. In vitro experiments confirmed that all compounds exhibited antiviral activity, albeit to varying degrees. Among them, vandetanib, nintedanib, dasatinib, avitinib, and nilotinib demonstrated relatively stronger efficacy. In vivo studies showed that these five kinase inhibitors were able to reduce viral load in mouse muscle tissue for a certain period, yet none significantly improved overall survival. In contrast, lycorine, as a direct‐acting antiviral agent, effectively suppressed viral replication and enhanced survival outcomes. Notably, vandetanib and avitinib delayed the onset of paralysis and ameliorated muscle pathology, indicating partial therapeutic benefits. The disparity in outcomes may be attributed to distinct mechanisms of action: lycorine directly inhibits viral protease activity, thereby blocking replication efficiently, whereas kinase inhibitors modulate host signaling pathways indirectly, potentially altering immune responses or viral‐host interactions without completely halting viral propagation. This indirect mechanism likely accounts for their more moderate antiviral effects and limited impact on survival and also helps explain the difference observed between strong in vitro efficacy and weaker in vivo outcomes. Additional factors may further contribute to this in vitro–in vivo discrepancy. Pharmacokinetic limitation, such as absorption, tissue penetration, metabolism, and clearance, could lower the effective drug concentration at the site of infection. Moreover, compensatory host signaling pathways may bypass kinase inhibition, whereas the immunomodulatory properties of these drugs could shift the balance between viral replication and inflammation. Together, these factors provide a plausible explanation for the divergence between in vitro and in vivo results. These findings also highlight a limitation of the present study: although host‐directed kinase inhibitors can ameliorate certain pathological features, none achieved robust therapeutic efficacy in vivo, underscoring the challenge of translating cell‐based antiviral activity into effective systemic treatments and suggesting that combination strategies, dosage optimization, or co‐administration with direct‐acting antivirals may be required.

## 4 Conclusions

We analyzed the molecular dynamics of EV‐A71 virus infection by using a multi‐omics method and generated a comprehensive transcriptomic, proteomic, and phosphoproteomic map of muscle tissue after virus infection. The drug targets of EV‐A71 were screened according to kinase activity and provide target information for identifying candidate therapeutic drugs. This study enhances our understanding of the biology of EV‐A71 and offers a foundation for further investigation into its pathogenic mechanism.

## 5 Materials and Methods

### 5.1 Cells and Drugs

RD cells (ATCC, Manassas, USA) were cultured at 37°C in a humidified atmosphere containing 5% CO_2_ in Dulbecco's Modified Eagle's Medium (DMEM; Gibco, Grand Island, USA) supplemented with 10% fetal bovine serum (FBS; Gibco) and 1% penicillin‐streptomycin (PS; Gibco). Regorafenib, zanubrutinib, sorafenib, vandetanib, pralsetinib, ponatinib, lenvatinib, selpercatinib, nintedanib, dasatinib, baricitinib, tofacitinib, acalabrutinib, avitinib, nilotinib, ellagic acid, and ATP were obtained from Selleck Chemicals (Houston, USA), whereas lycorine was purchased from Solarbio Science & Technology Co. Ltd. (Beijing, China).

### 5.2 Viruses

The mouse‐adapted EV‐A71 strain MP10 (GenBank: HQ712020), derived from FY0805 (GenBank: HQ882182), was used in BALB/c mice experiments [13], whereas the original FY0805 strain was used in RD cell infections. Neonatal BALB/c mice (7‐day‐old, *n* = 8 per group) were inoculated via intraperitoneal route with logarithmically serially diluted viral stock (10‐fold decrements). The 50% lethal dose (LD_50_) was subsequently determined through probit analysis of mortality kinetics monitored over a 14‐day observation period.

### 5.3 Mouse Experiments

Specific pathogen‐free (SPF) 7‐day‐old BALB/c mice were obtained from Beijing HFK Bioscience Ltd., and the mice weighed 3–4 g before viral challenge. Four mice from each group were inoculated i.p. with EV‐A71 at a dose of 100 LD_50_, and PBS was used as a control. Mice were sacrificed and dissected at 0, 2, and 5 dpi for muscle collection to evaluate virus replication, histopathological changes, and transcriptome profiles. Proteomic and phosphoproteomic analyses were performed on three mice in each group.

To investigate the role of NETs in viral infections, neutrophil knockout experiments were performed using 7‐day‐old BALB/c mice. The mice were injected i.p. with 100 µL 100 LD_50_ EV‐A71 virus and 2 h later i.p. with 10 mg/kg anti‐ly6G (100 µL, BioXcell, West Lebanon, USA). This was followed by administration at 1‐day intervals, with the rat IgG2a isotype control (10 mg/kg, BioXcell) as a control. Blood and muscle samples were collected at 5 dpi, and the viral loads were quantified (*n* = 6 per group); the mice were weighed daily, and their mortality, and clinical scores were monitored for 14 days (*n* = 8 per group). Clinical scoring rubric: health score was 0; 1 point for reduced activity; arched back, piloerection 2 points; single paralysis of hind limb 3 points; hind limb double limb palsy score 4 points; death counts as 5 points [43].

To test drug efficacy in vivo, SPF 10‐day‐old BALB/c mice were obtained from Beijing HFK Bioscience Ltd., and the mice weighed 5–6 g before inoculation. For lethal challenge, mice were i.p. injected with EV‐A71 at a dosage of 100 LD_50_ per 100 µL inoculum volume per mouse and 0.1 LD_50_ for sublethal dose infection (no mortality is observed in animals, not shown). Vandetanib (10 mg/kg/day, oral), nintedanib (5 mg/kg/day, oral), dasatinib (10 mg/kg/day, oral), avitinib (5 mg/kg/day, oral), nilotinib (5 mg/kg/day, i.p.), and lycorine (0.6 mg/kg/day, i.p.) were administered 2 h before infection and then daily. The control group was administered PBS orally each day in equivalent volumes. Body weight, mortality and clinical symptoms of the mice (*n* = 8 per group) were observed for 14 days following lethal challenge. In a parallel sublethal challenge, mice (*n* = 6 per group) were sacrificed at 5 dpi for blood and muscle collection to determine viral load and pathological changes.

### 5.4 Viral Inhibition Assay

RD cells were seeded into 96‐well plates in DMEM (10% FBS, 1% PS) 1 day before infection and were grown to a confluency of 80%. Two hours before infection, 100 µL of DMEM containing either the drugs of interest (regorafenib, zanubrutinib, sorafenib, vandetanib, pralsetinib, ponatinib, lenvatinib, selpercatinib, nintedanib, dasatinib, baricitinib, tofacitinib, acalabrutinib, avitinib, nilotinib, ellagic acid, ATP, and lycorine) or DMSO (control) was incubated with 100 µL of 100 times the 50% tissue culture infectious dose (TCID_50_) EV‐A71. The concentrations used were 0.032, 0.08, 0.32, 0.8, 1, 4, 8, and 32 µM. The medium was subsequently replaced with the mixture obtained in the previous step. The cells were incubated in an incubator for 72 h to observe the results. The cell supernatant was then collected to determine the viral load. All assays were performed in biologically independent triplicates.

### 5.5 Cytotoxicity Cell Viability Assays

Cytotoxicity was assessed using an MTS assay (Abcam) according to the manufacturer's instructions. Cytotoxicity was assessed in uninfected RD cells with the same compound dilutions as those used in the viral inhibition assay. All assays were performed in biologically independent triplicates.

### 5.6 Statistical Analysis

The viral load values are expressed as the mean ± SEM. Statistical analysis was performed between two groups using an unpaired two‐tailed Student's *t*‐test. For multiple group comparisons, one‐way analysis of variance (ANOVA) was performed. All the statistical analyses were performed using GraphPad Prism software version 9.

## Author Contributions

Conceptualization: Jiangning Liu, Juntao Yang, and Jiangfeng Liu. Methodology: Wanjun Peng, Qiaochu Wang, Binbin Zhao, Lihong Zhang, Jing Wu, Xiaohui Wei, Na Rong, Zhaohua Wang, and Kaihui Liu. Writing – original Draft: Wanjun Peng. Writing – review and editing: Jiangning Liu, Juntao Yang, and Jiangfeng Liu. Funding Acquisition: Jiangning Liu and Juntao Yang. Resources: Jiangning Liu and Juntao Yang. Supervision: Jiangning Liu and Juntao Yang. All authors read and approved the final manuscript.

## Funding

This work was supported by the National Key Research and Development Project of China (Grant No. 2022YFC2303404) and the CAMS Innovation Fund for Medical Sciences (CIFMS) grant 2022‐I2M‐1‐020, 2021‐1‐I2 M‐035, 2021‐I2M‐1‐044, 2022‐I2M‐2‐001, 2022‐I2M‐1‐011, 2021‐I2M‐1‐057, 2021‐I2M‐1‐049, S2021‐I2M‐1‐016 and S2021‐12M‐1‐001.

## Ethics Statement

Mouse studies were performed in an animal biosafety level 2 (ABSL‐2) facility using high‐efficiency particulate air (HEPA)‐filtered isolators. All animal procedures were reviewed and approved by the Institutional Animal Care and Use Committee of the Institute of Laboratory Animal Science, Peking Union Medical College (ILAS, PUMC, LJN23018).

## Conflicts of Interest

The authors declare no conflicts of interest.

## Supporting information




**Supporting Figure 1**: H&E staining and viral load detection for the muscle tissues of the control and infected BALB/c mice. (A–E) Skeletal muscle sections from PBS‐treated and EV‐A71‐infected mice were stained with H&E. Skeletal muscles of mice treated with PBS were collected at 0 dpi (A), 2 dpi (B), and 5 dpi (C); skeletal muscles of mice infected with EV‐A71 were collected at 2 dpi (D) and 5 dpi (E); Bar = 100 µm. (F) EV‐A71 viral loads in the blood and muscle tissues of BALB/c mice, *n* = 5.
**Supporting Figure 2**: Quality control (QC) and global profiling of the transcriptome, proteome, and phosphoproteome in the muscles of BALB/c mice. (A–C) PCA of quantifiable genes in the transcriptome (A), proteins in the proteome (B), and phosphosites in the phosphoproteome (C) of the muscle tissues. (D–F) The expression at each time point in the virus‐infected group was compared with that at the corresponding time point in the control group. Red represents significantly upregulated expression, blue represents significantly downregulated expression, and gray represents molecules with nonsignificant differences. The quantifiable and differentially expressed genes (D), proteins (E), phosphosites and their corresponding proteins (F) are shown. (G–I) Venn diagram of differentially expressed genes (G), proteins (H), and phosphorylation sites (I) between 2 and 5 dpi. (J–K) Venn diagram of the transcriptome, proteome, and phosphoproteome at 2 dpi (J) and 5 dpi (K).
**Supporting Figure 3**: Comprehensive Molecular Validation of Transcriptome and Proteome Profiles. Q‐PCR validation of transcriptome up‐(A) and down‐(B)regulated genes, *n* = 5. (C) WB validation of proteomic up‐ and down‐regulated proteins.
**Supporting Figure 4**: Workflow of Animal Experiments. (A) Neutrophil knockout animal experiment. (B) Kinase inhibitor drug efficacy testing experiment.
**Supporting Figure 5**: Flow cytometric analysis of neutrophil depletion in mice. (A–D) Representative flow cytometry plots showing neutrophils in blood (A and C) and muscle (B and D) at 1 day (A and B) and 5 days (C and D) post infection. (E and F) Quantification of neutrophil percentages in blood (E) and muscle (F), *n* = 3.
**Supporting Figure 6**: KEGG pathway enrichment in multi‐omics data of EV‐A71 infection. KEGG pathway enrichment of upregulated (red arrow) or downregulated (blue arrow) transcripts, proteins, and phosphorylation sites in the muscle tissues of EV‐A71‐infected mice at the indicated times after infection. The top 5 KEGG pathways in each group are plotted.
**Supporting Figure 7**: Drug toxicity profiling and kinase–inhibitor network analysis. (A–R) Drug toxicity test. An MTS assay was used to determine cell viability after the administration of different concentrations of drugs (*n* = 3). Regorafenib (A), zanubrutinib (B), sorafenib (C), vandetanib (D), pralsetinib (E), ponatinib (F), lenvatinib (G), selpercatinib (H), nintedanib (I), dasatinib (G), baricitinib (K), tofacitinib (L), acalabrutinib (M), avitinib (N), nilotinib (O), ellagic acid (P), ATP (Q), and lycorine (R). (S) Substrate–kinase–pathway–drug network in vandetanib and avitinib. Mapping regulated kinases to kinase inhibitors identifies EV‐A71 therapies.

**Supporting Table 1**: Differentially expressed proteins identified from the proteomic analysis.

**Supporting Table 2**: Functional analysis of differentially expressed genes, proteins and phosphosites in muscle tissues.
**Supporting Table 3**: Predicted kinase activity in the muscles of mice.
**Supporting Table 4**: Primer sequences list.
**Supporting Table 5**: Antibodies for Western Blot analysis.

## Data Availability

The original contributions presented in this study are included in the article/figure material, the RNA‐sequencing datasets generated for this study can be found in the Gene Expression Omnibus with accession nos. GSE252789, the mass spectrometry proteomics data have been deposited to the ProteomeXchange Consortium via the iProX partner repository with the dataset identifier PXD048236.
