# Supplementary material for: Systematic Proteomic Characterization of EV‐A71‐Infected Mice Identifies Dynamic Molecular Changes and Therapeutic Targets
Source: MedComm (2020). 2026 Apr 2;7(4):e70668. doi: 10.1002/mco2.70668 (PMC13052315; doi:10.1002/mco2.70668)
Supplement: Supplementary file 1 — Supporting Figure 1: H&E staining and viral load detection for the muscle tissues of the control and infected BALB/c mice. (A–E) Skeletal muscle sections from PBS‐treated and EV‐A71‐infected mice were stained with H&E. Skeletal muscles of mice treated with PBS were collected at 0 dpi (A), 2 dpi (B), and 5 dpi (C); skeletal muscles of mice infected with EV‐A71 were collected at 2 dpi (D) and 5 dpi (E); Bar = 100 µm. (F) EV‐A71 viral loads in the blood and muscle tissues of BALB/c mice, n = 5. Supporting Figure 2: Quality control (QC) and global profiling of the transcriptome, proteome, and phosphoproteome in the muscles of BALB/c mice. (A–C) PCA of quantifiable genes in the transcriptome (A), proteins in the proteome (B), and phosphosites in the phosphoproteome (C) of the muscle tissues. (D–F) The expression at each time point in the virus‐infected group was compared with that at the corresponding time point in the control group. Red represents significantly upregulated expression, blue represents significantly downregulated expression, and gray represents molecules with nonsignificant differences. The quantifiable and differentially expressed genes (D), proteins (E), phosphosites and their corresponding proteins (F) are shown. (G–I) Venn diagram of differentially expressed genes (G), proteins (H), and phosphorylation sites (I) between 2 and 5 dpi. (J–K) Venn diagram of the transcriptome, proteome, and phosphoproteome at 2 dpi (J) and 5 dpi (K). Supporting Figure 3: Comprehensive Molecular Validation of Transcriptome and Proteome Profiles. Q‐PCR validation of transcriptome up‐(A) and down‐(B)regulated genes, n = 5. (C) WB validation of proteomic up‐ and down‐regulated proteins. Supporting Figure 4: Workflow of Animal Experiments. (A) Neutrophil knockout animal experiment. (B) Kinase inhibitor drug efficacy testing experiment. Supporting Figure 5: Flow cytometric analysis of neutrophil depletion in mice. (A–D) Representative flow cytometry plots showing neutrophils in [file MCO2-7-e70668-s003.docx]

**Systematic Proteomic Characterization of EV-A71-Infected Mice Identifies Dynamic Molecular Changes and Therapeutic Targets**

**Running title:** Proteomic in EV-A71-infected mice

**Wanjun Peng^1, 2^, Qiaochu Wang^2^, Binbin Zhao^1^, Lihong Zhang^1^, Jing Wu^1^, Xiaohui Wei^1^, Na Rong****^1^, Zhaohua Wang^1^, Kaihui Liu^1^, Jiangfeng Liu****^2^*, Juntao Yang^2^*, Jiangning Liu^1^***

^1^NHC Key Laboratory of Human Disease Comparative Medicine, Beijing Key Laboratory for Animal Models of Emerging and Remerging Infectious Diseases, Institute of Laboratory Animal Science, Chinese Academy of Medical Sciences and Comparative Medicine Center, Peking Union Medical College, Beijing, China.

^2^State Key Laboratory of Common Mechanism Research for Major Diseases, Department of Biochemistry and Molecular Biology, Institute of Basic Medical Sciences Chinese Academy of Medical Sciences, School of Basic Medicine Peking Union Medical College, Beijing, 100005 China

Wanjun Peng, and Qiaochu Wang contributed equally to this work.

Jiangfeng Liu, Juntao Yang and Jiangning Liu are corresponding authors to this work

* Corresponding author

Jiangfeng Liu *E-mail: ljf@pumc.edu.cn, [liujiangf@126.com](mailto:liujiangf@126.com)*

Juntao Yang *E-mail:* *yangjt@pumc.edu.cn*

Jiangning Liu *E-mail: [liujn@cnilas.org](mailto:liujn@cnilas.org)*

***Supplementary material***

***Supplementary Figure***

**
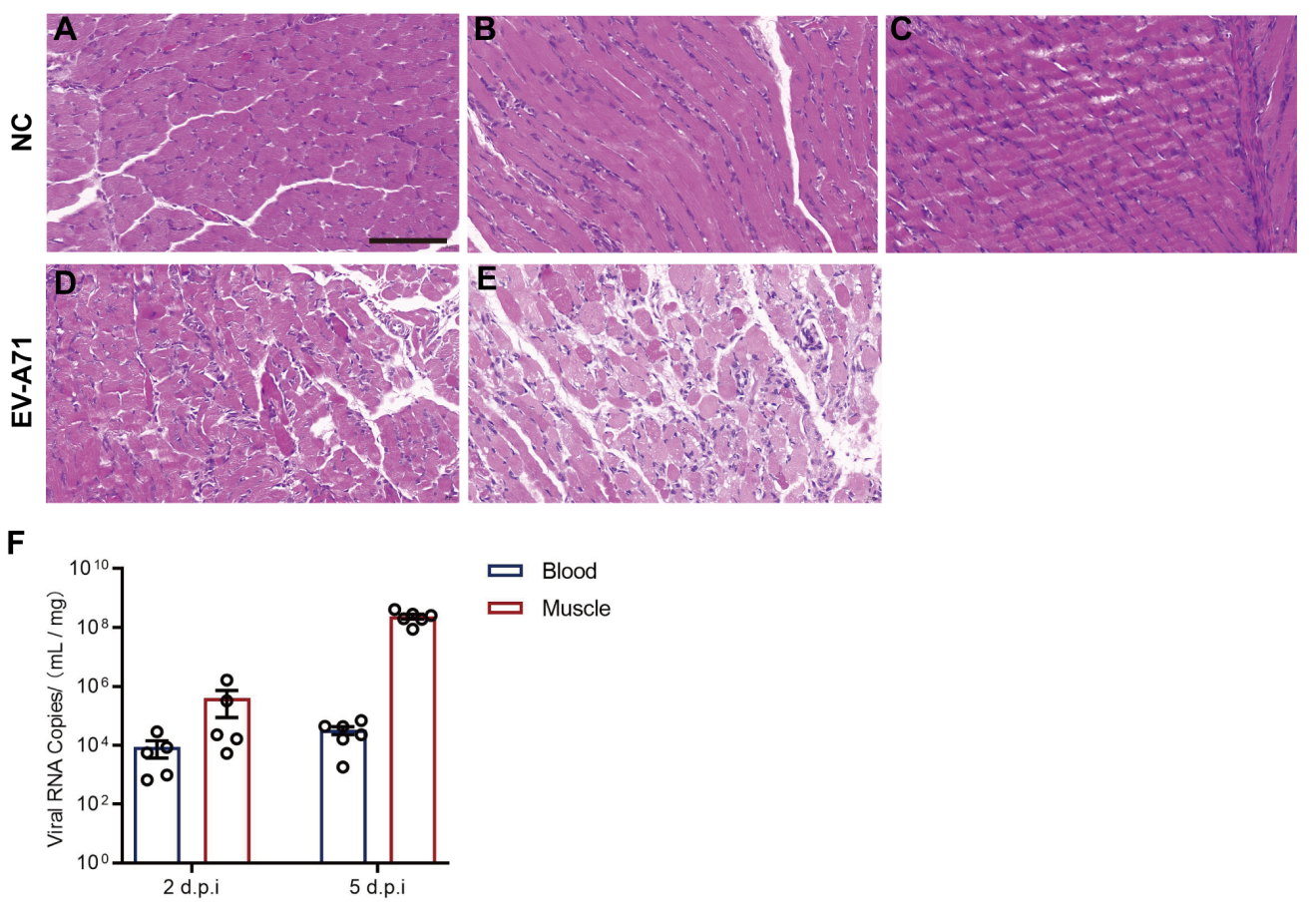
**

**Supplementary Figure S1** H&E staining and viral load detection for the muscle tissues of the control and infected BALB/c mice. (A-E) Skeletal muscle sections from PBS-treated and EV-A71-infected mice were stained with H&E. Skeletal muscles of mice treated with PBS were collected at 0 dpi (A), 2 dpi (B) and 5 dpi (C); skeletal muscles of mice infected with EV-A71 were collected at 2 dpi (D) and 5 dpi (E); Bar = 100 μm. (F) EV-A71 viral loads in the blood and muscle tissues of BALB/c mice, n = 5.


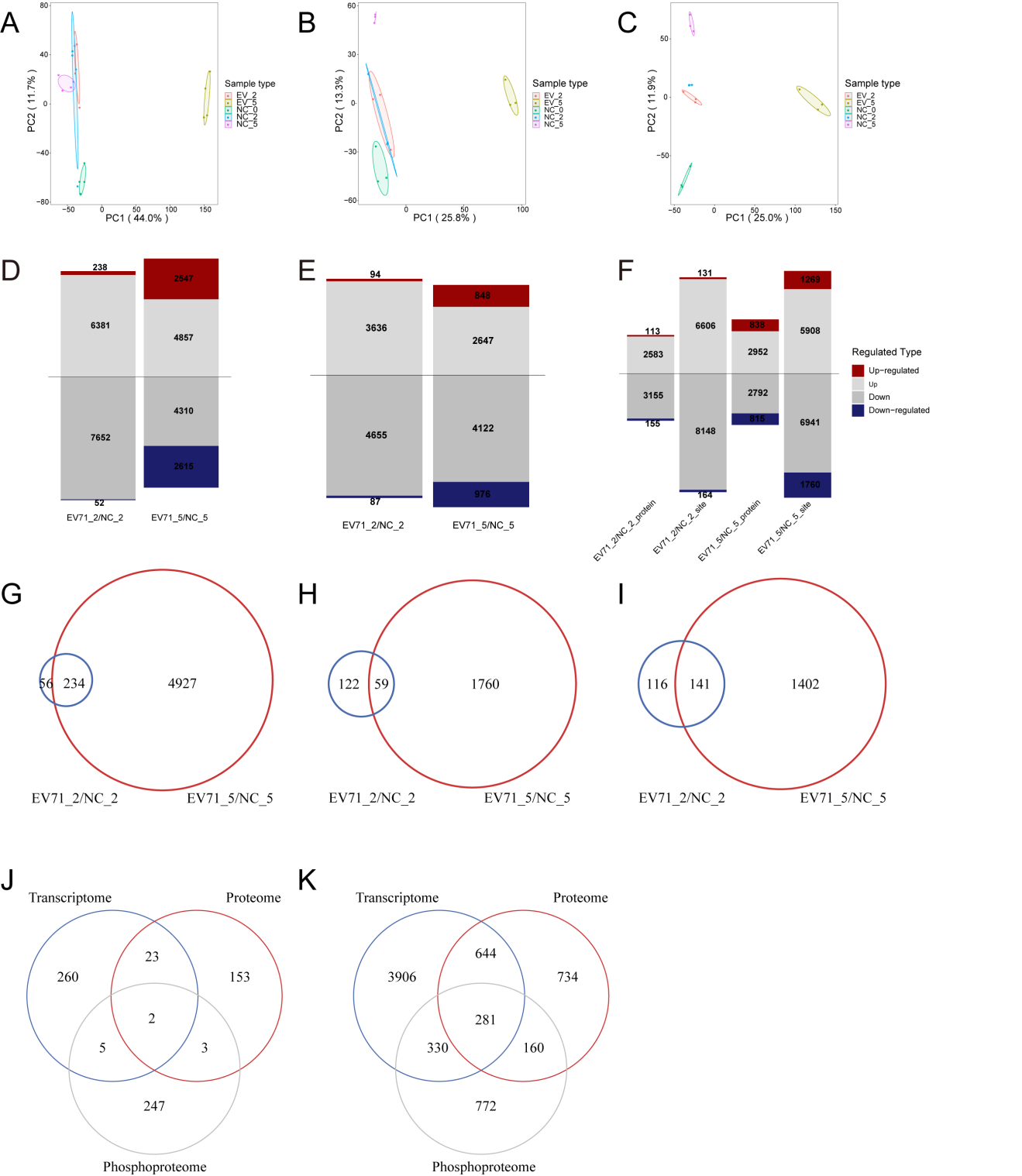


**Supplementary Figure S2** Quality control (QC) and global profiling of the transcriptome, proteome and phosphoproteome in the muscles of BALB/c mice. (A-C) PCA of quantifiable genes in the transcriptome (A), proteins in the proteome (B) and phosphosites in the phosphoproteome (C) of the muscle tissues. (D-F) The expression at each time point in the virus-infected group was compared with that at the corresponding time point in the control group. Red represents significantly upregulated expression, blue represents significantly downregulated expression, and gray represents molecules with nonsignificant differences. The quantifiable and differentially expressed genes (D), proteins (E), phosphosites and their corresponding proteins (F) are shown. (G-I) Venn diagram of differentially expressed genes (G), proteins (H) and phosphorylation sites (I) between 2 dpi and 5 dpi. (J-K) Venn diagram of the transcriptome, proteome and phosphoproteome at 2 dpi (J) and 5 dpi (K).


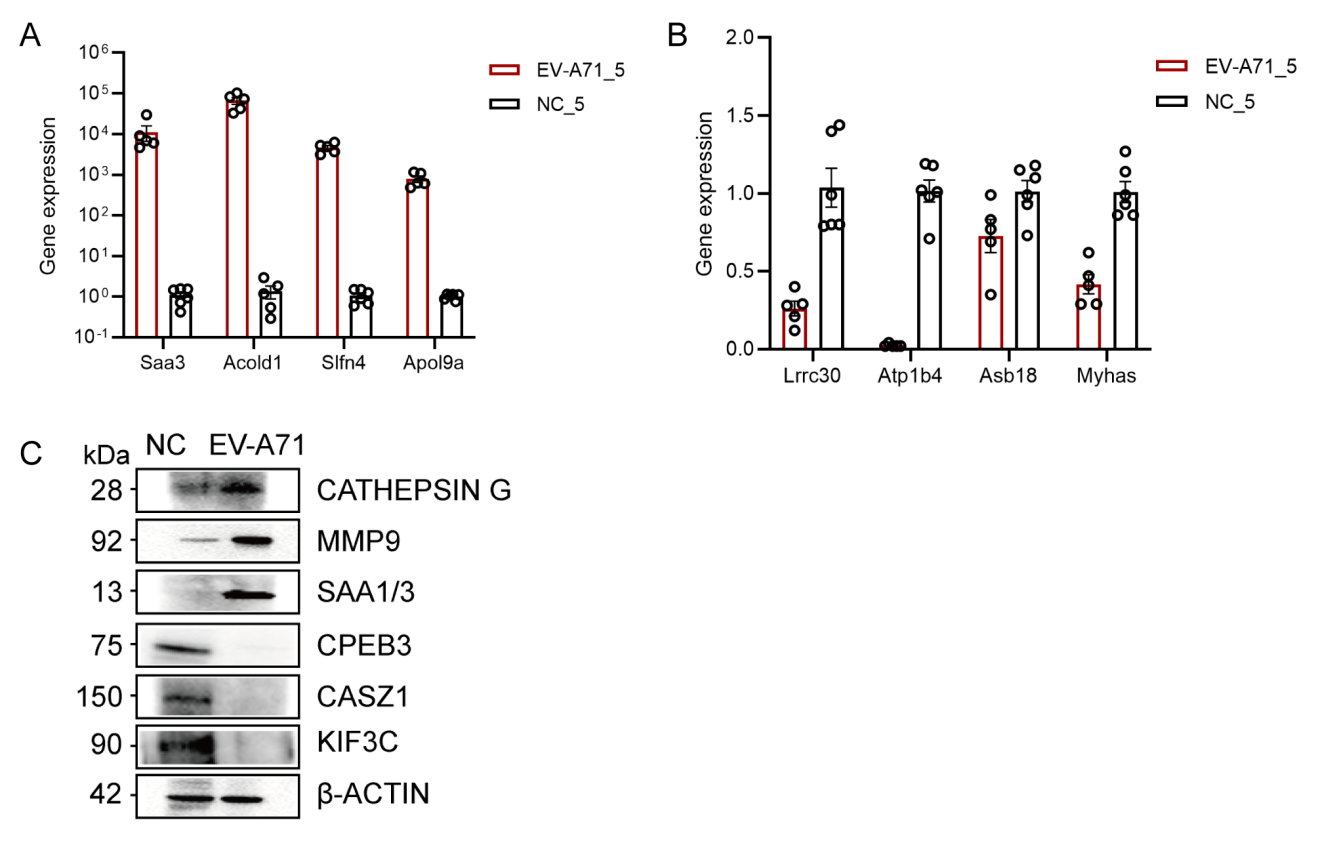


**Supplementary Figure S3** Comprehensive Molecular Validation of Transcriptome and Proteome Profiles. Q-PCR validation of transcriptome up-(A) and down-(B)regulated genes, n = 5. (C) WB validation of proteomic up- and down-regulated proteins.


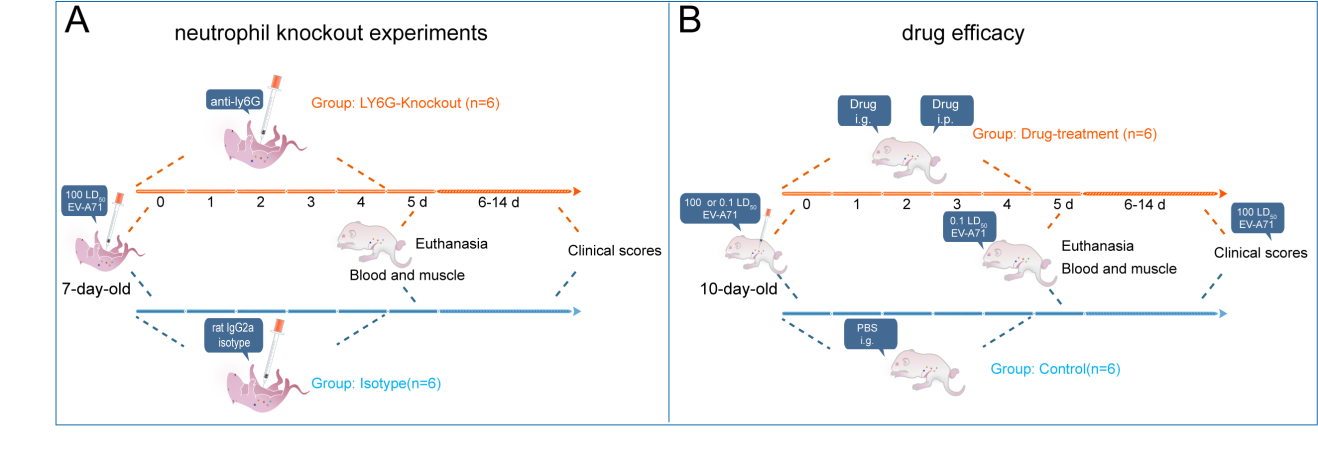


**Supplementary Figure S4** Workflow of Animal Experiments. (A) Neutrophil knockout animal experiment. (B) Kinase inhibitor drug efficacy testing experiment.


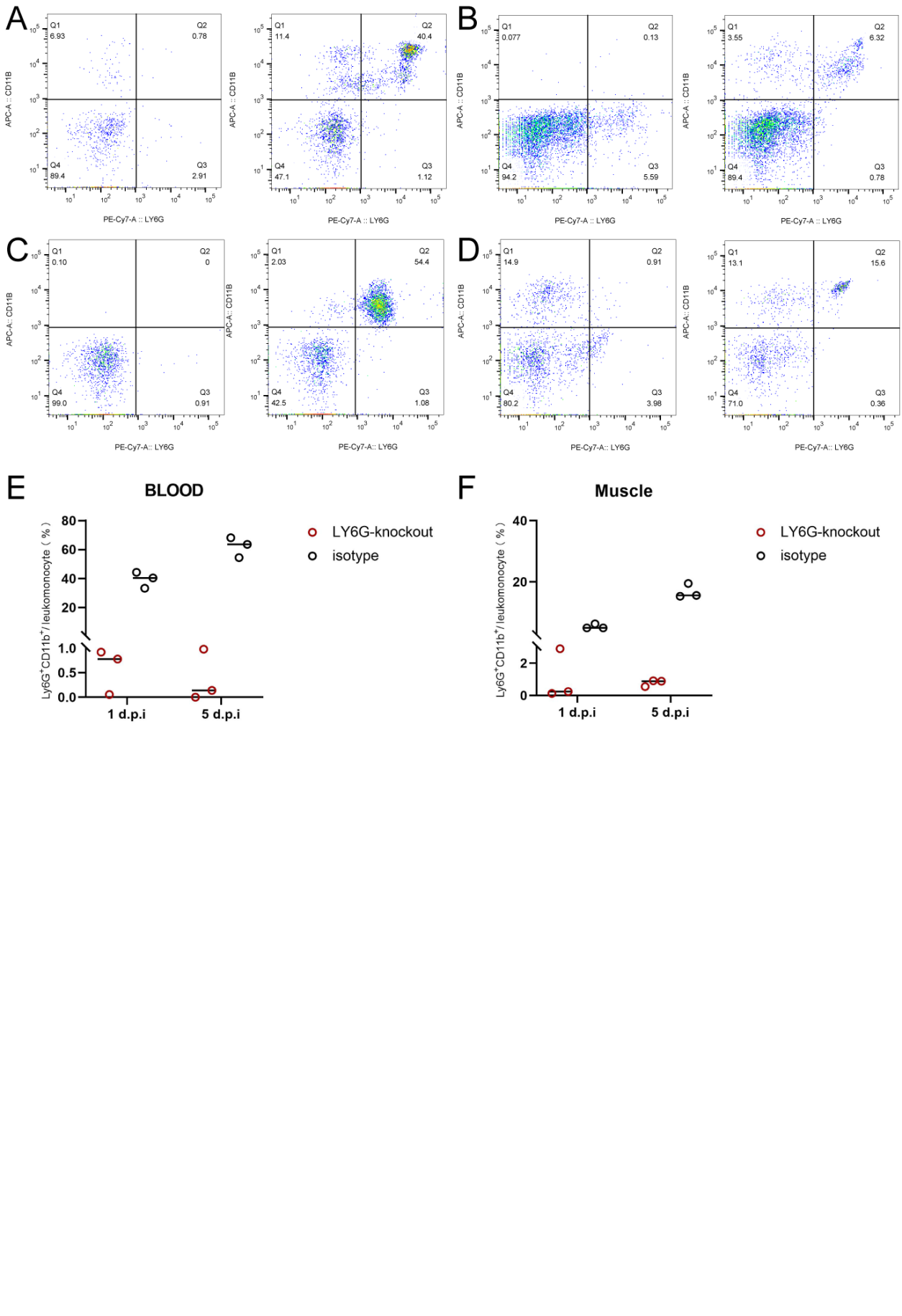


**Supplementary Figure S5** Flow cytometric analysis of neutrophil depletion in mice. (A–D) Representative flow cytometry plots showing neutrophils in blood (A, C) and muscle (B, D) at 1 day (A, B) and 5 days (C, D) post infection. (E, F) Quantification of neutrophil percentages in blood (E) and muscle (F), n = 3.

**
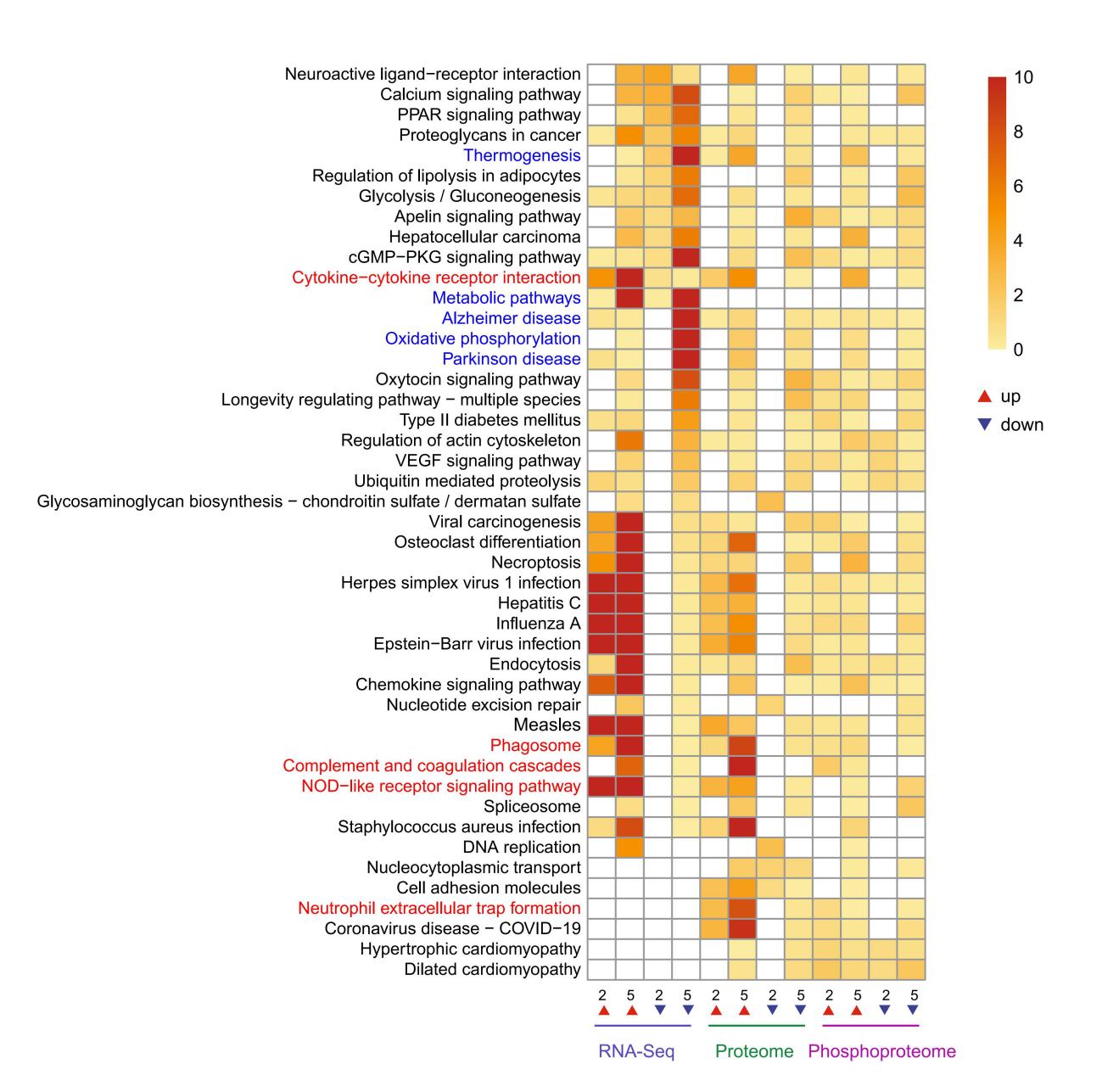
**

**Supplementary Figure S6** KEGG pathway enrichment in multiomics data of EV-A71 infection. KEGG pathway enrichment of upregulated (red arrow) or downregulated (blue arrow) transcripts, proteins and phosphorylation sites in the muscle tissues of EV-A71-infected mice at the indicated times after infection. The top 5 KEGG pathways in each group are plotted.

**
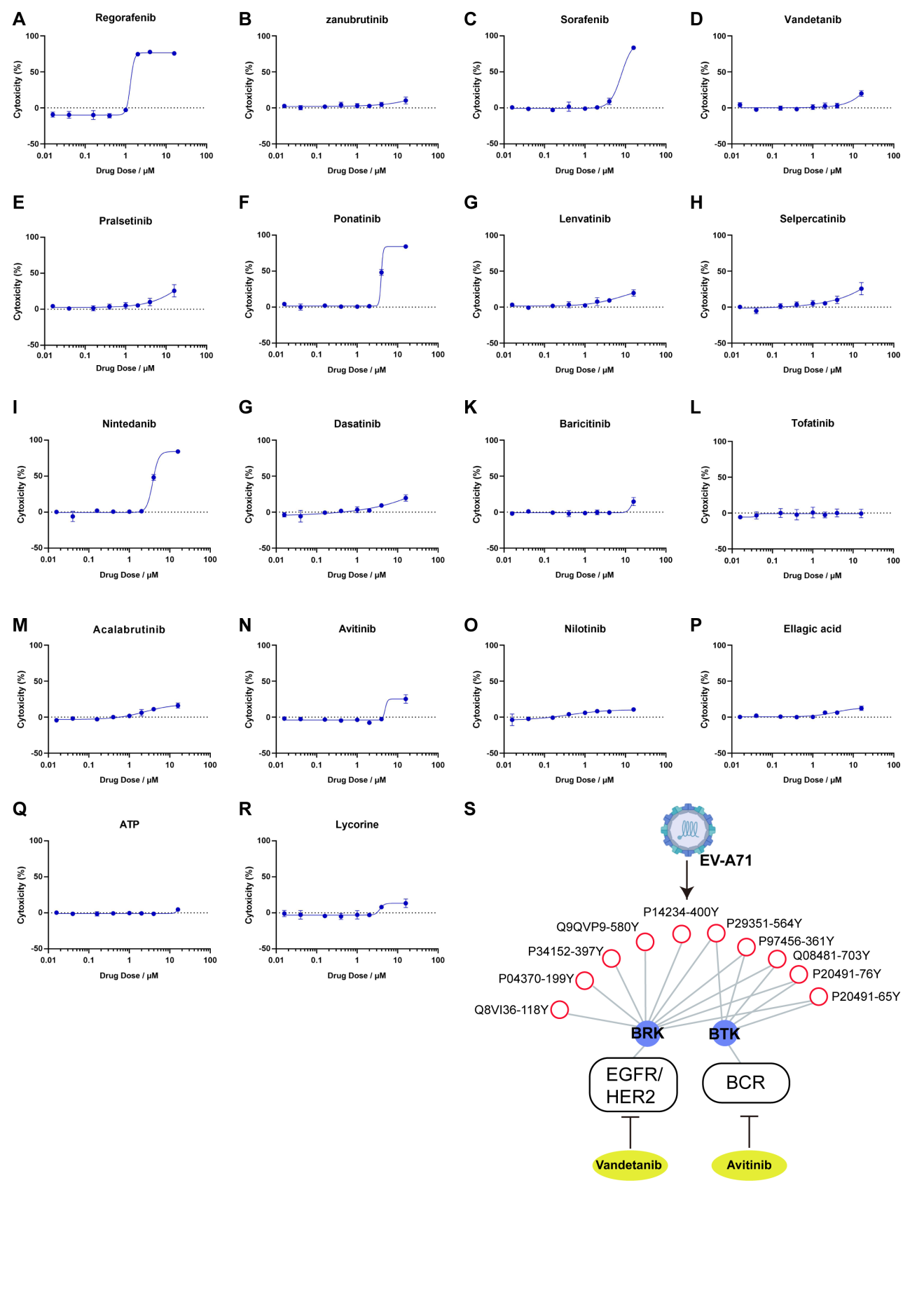
**

**Supplementary Figure S7** Drug toxicity profiling and kinase–inhibitor network analysis. (A-R) Drug toxicity test. An MTS assay was used to determine cell viability after the administration of different concentrations of drugs (n=3). Regorafenib (A), zanubrutinib (B), sorafenib (C), vandetanib (D), pralsetinib (E), ponatinib (F), lenvatinib (G), selpercatinib (H), nintedanib (I), dasatinib (G), baricitinib (K), tofacitinib (L), acalabrutinib (M), avitinib (N), nilotinib (O), ellagic acid (P), ATP (Q) and lycorine (R). (S) Substrate–kinase–pathway–drug network in vandetanib and avitinib. Mapping regulated kinases to kinase inhibitors identifies EV-A71 therapies.

***Supplementary Table***

**Supplementary Table 1** Differentially expressed proteins identified from the proteomic analysis.

**Supplementary Table 2** Functional analysis of differentially expressed genes, proteins and phosphosites in muscle tissues.

**Supplementary Table 3** Predicted kinase activity in the muscles of mice.

|  | EV0vsNC0 | EV2vsNC2 | EV5vsNC5 |
| --- | --- | --- | --- |
| ABL1 | 0 | 1.1053872 | 1.4493513 |
| ABL2 | 0 | 1.1172097 | 1.4755876 |
| AMPKA1 | 0 | -0.65866 | -1.3906451 |
| BARK1 | 0 | -1.1841433 | 1.3644751 |
| BRK | 0 | -1.3331633 | 1.7891792 |
| BTK | 0 | -1.2411468 | 1.8123127 |
| CAMK1G | 0 | 1.4998066 | -0.93473023 |
| CK1D | 0 | -1.0163456 | -1.3396512 |
| CK1E | 0 | -1.0916231 | -1.4719577 |
| CK2A2 | 0 | -1.2616764 | 1.2524848 |
| DCLK2 | 0 | 1.2749082 | 1.2646725 |
| ERBB2 | 0 | -1.0900998 | 1.6168767 |
| ERBB3 | 0 | -1.092826 | 1.6040446 |
| FAK | 0 | -1.0789448 | 1.633333 |
| FER | 0 | -0.9185325 | 1.7136343 |
| FES | 0 | 0.73099214 | 1.7280803 |
| FRK | 0 | -0.88829595 | 1.7731448 |
| FYN | 0 | -1.0732945 | 1.9106445 |
| ITK | 0 | -1.4403415 | 1.4227232 |
| JAK2 | 0 | 0.84798366 | 1.480175 |
| JAK3 | 0 | 0.8678085 | 1.4984858 |
| LATS1 | 0 | 1.5156397 | 1.2611908 |
| LATS2 | 0 | 1.1525135 | 1.634188 |
| LCK | 0 | -1.1163429 | 1.8459986 |
| LYN | 0 | -1.3550493 | 1.7920362 |
| MAPKAPK2 | 0 | -0.8761433 | 1.3950562 |
| MAST1 | 0 | 1.5150516 | 1.2515599 |
| MAST3 | 0 | 1.5179098 | 1.257814 |
| MAST4 | 0 | 1.5140438 | 1.254714 |
| NDR1 | 0 | 1.5276606 | 1.3365337 |
| NEK10 | 0 | 1.4672586 | -0.8142053 |
| NEK11 | 0 | 1.5596901 | -0.80494225 |
| NEK3 | 0 | 1.5319704 | -0.8158255 |
| NEK5 | 0 | 1.5300382 | -0.82319844 |
| NEK6 | 0 | 1.5497373 | -0.8129034 |
| NEK7 | 0 | 1.5150045 | -0.8134384 |
| NEK8 | 0 | 1.4251738 | 0.77362335 |
| NEK9 | 0 | 1.5382217 | -0.80125463 |
| PIM3 | 0 | 1.2735586 | 1.2272145 |
| PKD1 | 0 | -0.7880019 | 1.5878736 |
| PKD2 | 0 | -0.78806907 | 1.566862 |
| PKN1 | 0 | 1.1538161 | 1.6285622 |
| PSKH1 | 0 | 1.1527052 | 1.237024 |
| PYK2 | 0 | -0.78912854 | 1.4840071 |
| RET | 0 | -1.2330534 | 1.4866327 |
| RSKL1 | 0 | 1.5281026 | 1.2597016 |
| RSKL2 | 0 | 1.5227125 | 1.2669433 |
| SGK2 | 0 | 1.3317846 | 1.4477544 |
| SGK494 | 0 | 1.5332923 | 1.2630117 |
| SSTK | 0 | 1.2432556 | 1.2641373 |
| STK33 | 0 | 1.1992071 | 1.2614682 |
| SYK | 0 | -0.84694684 | 1.782314 |
| TSSK3 | 0 | 1.2732346 | 1.2692921 |
| TSSK4 | 0 | 1.2875681 | 1.2318295 |
| TSSK5 | 0 | 1.276119 | 1.2320508 |
| TTBK1 | 0 | -1.0337437 | -1.3482552 |
| VACAMKL | 0 | 1.2201134 | 1.2345918 |
| WNK1 | 0 | -0.6194258 | 1.4992161 |
| YANK2 | 0 | 1.5447361 | 1.2649826 |
| YANK3 | 0 | 1.5207431 | 1.2516919 |
| YES | 0 | -0.88127714 | 1.7861668 |
| ZAP70 | 0 | -1.0603673 | 1.7347662 |

**Supplementary Table 4** Primer sequences list.

| Gene | Sequences |
| --- | --- |
| EV-A71-F | GCAGCCCAAAAGAACTTCACT |
| EV-A71-R | ATCTGCCACCCTATCTCCCT |
| EV-A71-probe | FAM-TGCAAGGATGCTAGTGATATCCTGC-TAM |
| Myhas-F | CCCGTTTCTGGAGGGTTT |
| Myhas-R | CATGGTGGCCGATCATTT |
| Lrrc30-F | CAGGCAGGACCAACATCTAAC |
| Lrrc30-R | GCAGGGTATCATCCCACGAC |
| Atp1b4-F | CCTCCTCAAGATGAACCG |
| Atp1b4-R | GGTAAAGATTATCCTCCCAAC |
| Asb18-F | TGAGGTGGAGCCTGTGGA |
| Asb18-R | TTTGCGACTGAGCAGGTGT |
| Gapdh-F | AATGTGTCCGTCGTGGATCT |
| Gapdh-R | CATCGAAGGTGGAAGAGTGG |
| Saa3-F | CGCAGCACGAGCAGGAT |
| Saa3-R | TGGCTGTCAACTCCCAGG |
| Slfn4-F | GCCCTCTGTTCAAGTCAAGTGTCC |
| Slfn4-R | CCCAGATGAAATCCTTTCCACGA |
| Apol9a-F | TCTGACATCCTGAGCCTCCTTGG |
| Apol9a-R | GCCAGTCGGAGCAGCTTCAAC |
| Acod1-F | GGCACAGAAGTGTTCCATAAAGT |
| Acod1-R | GAGGCAGGGCTTCCGATAG |

**Supplementary Table 5 Antibodies for Western Blot Analysis**.

| **Antibody Name** | **Dilution** | **Supplier** | **Catalog No.** |
| --- | --- | --- | --- |
| Jak3 Rabbit mAb | 1:1000 | Cell Signaling Technology (CST) | Cat#: 8863 |
| c-Abl Antibody | 1:1000 | CST | Cat#: 2862 |
| Jak2 Rabbit mAb | 1:1000 | CST | Cat#: 3230 |
| Ret Rabbit mAb | 1:1000 | CST | Cat#: 14556 |
| Lyn Rabbit mAb | 1:1000 | CST | Cat#: 2796 |
| Lck Antibody | 1:1000 | CST | Cat#: 2752 |
| Yes Rabbit mAb | 1:1000 | CST | Cat#: 65890 |
| Pyk2 Antibody | 1:1000 | CST | Cat#: 3292 |
| Btk Rabbit mAb | 1:1000 | CST | Cat#: 8547 |
| PTK6/BRK Rabbit mAb | 1:1000 | CST | Cat#: 55174 |
| Syk Rabbit mAb | 1:1000 | CST | Cat#: 13198T |
| PKD/PKC Rabbit mAb | 1:1000 | CST | Cat#: 90039 |
| Frk Polyclonal Antibody | 1:1000 | Immunoway | Cat#: YT3996 |
| Anti-CASZ1 | 1:1000 | Abcam | Cat#: ab259847 |
| Anti-MMP9 | 1:1000 | Abcam | Cat#: ab283575 |
| Anti-Cpeb3 | 1:1000 | Abcam | Cat#: ab10883 |
| Anti-Saa1/3 | 1:1000 | Abcam | Cat#: ab199030 |
| Anti-Kif3c | 1:1000 | Abcam | Cat#: ab236748 |
| Anti-Cathepsin G | 1:1000 | Affinity Biosciences | Cat#: AF5167 |
| Anti-GAPDH | 1:10000 | Abcam | Cat#: ab181602 |
| Anti-β-Actin | 1:1000 | Immunoway | Cat#: YT0099 |

***Supplementary Materials and methods***

1. **RNA extraction and quantitative real-time PCR**

RNA was extracted from mouse blood and muscle using TRIzol reagent (Invitrogen, Carlsbad, USA). Quantitative real-time PCR was performed using QuantiTect Probe RT–PCR Kit (Qiagen, Hilden, Germany) and One Step TB Green PrimeScript RT-PCR Kit (Takara, Kusatsu, Japan) according to the manufacturer's instructions. The viral load of EV-A71 was quantified using the standard curve method. The standard curve was generated by a series of tenfold dilutions of a recombinant plasmid of known concentration. The samples were processed using the following protocol and primers: 50 °C for 30 min and 95 °C for 15 min, followed by 45 cycles of 94 °C for 15 s and 60 °C for 1 min. Transcriptome gene validation was conducted using the 2-ΔΔCT method, with GAPDH serving as the internal reference gene. The samples were processed using the following protocol and primers: 42 °C for 5 min and 95 °C for 10 s, followed by 40 cycles of 95 °C for 5 s and 60 °C for 34 s, and 95 °C for 15 s, 60 °C for 1 min and 95 °C for 15 s. The list of primers is presented in Table S4.

1. **RNA sequencing**

The RNA-seq and bioinformatic analyses of this study were performed and analyzed by Beijing Nuohe Zhiyuan Technology. First, RNA was extracted and its quality was determined, the total amounts and integrity of RNA were evaluated using the RNA Nano 6000 Assay Kit of the Bioanalyzer 2100 system (Agilent Technologies, CA, USA). After quality inspection, a common eukaryotic transcriptome library was constructed by the magnetic bead enrichment method (NEBNext Ultra RNA Library Prep Kit for Illumina). Following quality control, libraries were constructed using the NEBNext Ultra RNA Library Prep Kit (Illumina) and quantified using a Qubit 2.0 Fluorometer. Libraries were sequenced on an Illumina NovaSeq 6000 platform (150 bp paired-end reads). Data were processed using CASAVA, aligned to the reference genome using Hisat2 (v2.0.5), and gene counts were obtained with FeatureCounts (v1.5.0-p3). Differentially expressed genes (DEGs) were identified using DESeq2 (p < 0.05, |FC| > 2).

**3. Pathological examination**

Skeletal muscles were fixed in 10% formalin for 72 h, dehydrated by alcohol gradient and embedded in paraffin. Hematoxylin and eosin staining was used to identify histopathological changes in paraffin sections of the tissues (5 μm thickness), which were observed via light microscopy. Each tissue sample was scored on a 0-5 scale based on lesion severity and extent. Scoring was performed blindly by two independent pathologists, and the average score was recorded. The pathology scoring rubric was as follows: 0, normal; 1, minimal change exceeding the normal range; 2, mild but observable lesion; 3, moderate lesion; 4, severe lesion involving most tissues and organs; 5, extensive lesion occupying the entire tissue or organ.

**4. Confocal microscopy**

Paraffin sections of muscle were deparaffinized in xylene, hydrated in ethanol, thermally repaired in alkaline antigenic repair solution and cooled to room temperature. Sections were then blocked in 10% goat serum for 10 min and stained with an anti-Myeloperoxidase monoclonal antibody (1:100, Abcam, Cambridge, UK) at room temperature for 1 hour. After incubation with HRP-labeled goat anti-rabbit IgG secondary antibody for 10 min, the sections were stained with PPD520. The above steps were repeated to stain with the antibodies Histone H3 (1:2000, abcam) and PPD570 was used to express the fluorescent signal. For DNA detection, DAPI was used. Sections were observed via fluorescence microscopy.

**5. Protein extraction and Western blotting**

Muscle tissue samples were placed in 1.5 ml centrifuge tubes, and protein cleavage buffer (1% Phosphatase inhibitor, 1% PMSF, Solarbio) was added. The tissue was homogenized using a grinder, and the supernatant was collected. A BCA Protein Assay Kit (Abcam) was used to determine the total protein concentration, and 10 μg of protein was subjected to SDS‒PAGE on a 4‒15% precast SDS-PAGE gel (Beyotime, Shanghai, China) and transferred to a nitrocellulose membrane. The membrane was blocked at room temperature for 1 h in blocking solution (Beyotime), and the membrane was incubated with the corresponding primary antibody at 4 °C overnight (Table S5). After the membranes were washed with TBST, they were incubated with a secondary antibody at room temperature for 1 h. Chemiluminescence detection was performed using an enhanced chemiluminescence (ECL) detection reagent (Solarbio), and the bands were imaged using a Bio-Rad ChemiDoc.

1. **Protein preparation and LC‒MS/MS analysis**

Approximately 100 mg of muscle tissue was placed in a 1.5 ml centrifuge tube, 4 volumes of lysis buffer (1% SDS, 1% protease inhibitor and 1% phosphatase inhibitor) were added, and the mixture was homogenized. The tissue homogenate was heated at 100 °C for 30 min (for virus inactivation) and centrifuged at 4 °C for 10 min at 12000 × g to remove protein fragments. The supernatant was collected for protein concentration detection by BCA assay. Then, 1 volume of precooled acetone was added to the protein sample, the mixture was vortexed, and 4 volumes of precooled acetone were added. The mixture was precipitated at -20 °C for 2 h. The precipitate was subsequently washed 2-3 times with precooled acetone. The protein sample was then redissolved in 200 mM TEAB and ultrasonically dispersed. Trypsin was added at 1:50 trypsin: protein mass ratio for the first digestion overnight, and the sample was then centrifuged at room temperature at 14000 × *g* for 20 min. The supernatant was collected for the second digestion (the mass ratio of trypsin: protein was 1:100), digested at 37 ℃ for 4 h, and centrifuged at room temperature at 14000 × *g* for 20 min. The supernatant was again collected and added to 20% trifluoroacetic acid, the pH was adjusted to 1-2, the sample was centrifuged at 12000 × g at room temperature for 10 min, and the supernatant was collected and desalted using an HLB demineralizer column. After the column was activated with methanol, 2 ml of ddH_2_O was slowly added, after which the supernatant was added, and the effluent was collected once. After washing twice with 5% methanol, the peptide on the desalting column was eluted with 1 ml of methanol, and the eluate was collected. The desalted peptide samples were evaporated to dryness using a vacuum rotary evaporator. An Agilent 300 Extend C18 column (5 μm particles, 4.6 mm ID, and 250 mm length) was used for high-pH reverse-phase HPLC to fractionate the peptides.

1. **Statistical analysis**

The false discovery rate (FDR) in RNA sequencing was regulated by adjusting the *P* values through Benjamini and Hochberg's method. *P* values < 0.05 were considered to indicate statistical significance. **P* < 0.05.

The peptide used for phosphoproteome analysis was incubated with an Fe IMAC microsphere suspension to enrich the phosphorylated peptide. After removing the nonspecific peptide, the Fe IMAC microspheres were washed with elution buffer. The elution supernatant containing the enriched phosphopeptides was collected and divided into different components for lyophilization.

An Orbitrap Exploris 480 mass spectrometer equipped with a FAMIS device and an EASY-nLC 1200 UPLC system were used for LC–MS/MS analysis. Tandem mass spectrometry (TMS) and data-dependent analysis (DDA) were used to identify and quantify peptides, proteins and phosphosites[1].

The original MS/MS raw files were submitted to MaxQuant (version 1.6.15.0) for database retrieval, and the basic data rearrangement was performed using Perseus 5 (version 1.6.5.0), including normalization and imputation. Student's t test was used for differential expression analysis, and *P* < 0.05 was considered to indicate statistical significance. The FC in total protein and of phosphorylation at various sites at different time points after infection was calculated (mean value of the virus-infected group/mean value of the control group). Upregulated molecules were defined as those with FC > 2, while downregulated molecules were defined as those with FC < 0.5. The quantified features at 0, 2, and 5 dpi (proteins for proteomics and phosphosites for phosphoproteomics) were standardized and analyzed for differences from the corresponding controls, followed by fuzzy C-means clustering based on FC values[2]. Proteins in each cluster were submitted to the STRING database for PPI analysis, and PPI networks were constructed using Cytoscape software[3]. Pathway functional enrichment analysis was performed using the KEGG database (Fisher's exact test; Benjamini–Hochberg's FDR correction method). Pathways with *P* values < 0.05 were defined as enriched. Kinase prediction was performed using iGPS1.0[4]. Kinase activity was calculated via GSEA and is presented as a heatmap[5]. Kinases with significantly altered activity were defined as those with a GSEA *P* value < 0.05 and | NES | > 1. DrugBank (www.drugbank.ca) is a web-enabled database containing comprehensive molecular information about drugs, their mechanisms, their interactions and their targets[6]. To identify FDA-approved targeted therapies for kinases with significantly altered activities, the DrugBank database was queried to extract drug-kinase target relationships. These data were then used to construct a Sankey diagram illustrating the connections between the kinases and their corresponding drugs.

Bioinformatic analysis, including PCA, volcano plots, and Venn diagrams, was performed using R software.

**References**

1. Liu J, Peng W, Wu Y et al. Proteomic and phosphoproteomic characteristics of the cortex, hippocampus, thalamus, lung, and kidney in COVID-19-infected female K18-hACE2 mice. *EBioMedicine*. 2023;90:104518

2. Zhang Y, Han J. Differential privacy fuzzy C-means clustering algorithm based on gaussian kernel function. *PLoS One*. 2021;16(3):e0248737

3. Shannon P, Markiel A, Ozier O et al. Cytoscape: a software environment for integrated models of biomolecular interaction networks. *Genome Res*. 2003;13(11):2498-504

4. Song C, Ye M, Liu Z et al. Systematic analysis of protein phosphorylation networks from phosphoproteomic data. *Mol Cell Proteomics*. 2012;11(10):1070-83

5. Subramanian A, Tamayo P, Mootha VK et al. Gene set enrichment analysis: a knowledge-based approach for interpreting genome-wide expression profiles. *Proc Natl Acad Sci U S A*. 2005;102(43):15545-50

6. Wishart DS, Feunang YD, Guo AC et al. DrugBank 5.0: a major update to the DrugBank database for 2018. *Nucleic Acids Res*. 2018;46(D1):D1074-D1082
